# Zika virus-like particle vaccine fusion loop mutation increases production yield but fails to protect AG129 mice against Zika virus challenge

**DOI:** 10.1371/journal.pntd.0010588

**Published:** 2022-07-06

**Authors:** Danielle Thompson, Ben Guenther, Darly Manayani, Jason Mendy, Jonathan Smith, Diego A. Espinosa, Eva Harris, Jeff Alexander, Lo Vang, Christopher S. Morello

**Affiliations:** 1 Emergent BioSolutions Inc., Gaithersburg, Maryland, United States of America; 2 PaxVax Inc., San Diego, California, United States of America; 3 Division of Infectious Diseases and Vaccinology, School of Public Health, University of California, Berkeley, Berkeley, California, United States of America; INSERM, FRANCE

## Abstract

Zika virus (ZIKV) is a mosquito-borne flavivirus with maternal infection associated with preterm birth, congenital malformations, and fetal death, and adult infection associated with Guillain-Barré syndrome. Recent widespread endemic transmission of ZIKV and the potential for future outbreaks necessitate the development of an effective vaccine. We developed a ZIKV vaccine candidate based on virus-like-particles (VLPs) generated following transfection of mammalian HEK293T cells using a plasmid encoding the pre-membrane/membrane (prM/M) and envelope (E) structural protein genes. VLPs were collected from cell culture supernatant and purified by column chromatography with yields of approximately 1-2mg/L. To promote increased particle yields, a single amino acid change of phenylalanine to alanine was made in the E fusion loop at position 108 (F108A) of the lead VLP vaccine candidate. This mutation resulted in a modest 2-fold increase in F108A VLP production with no detectable prM processing by furin to a mature particle, in contrast to the lead candidate (parent). To evaluate immunogenicity and efficacy, AG129 mice were immunized with a dose titration of either the immature F108A or lead VLP (each alum adjuvanted). The resulting VLP-specific binding antibody (Ab) levels were comparable. However, geometric mean neutralizing Ab (nAb) titers using a recombinant ZIKV reporter were significantly lower with F108A immunization compared to lead. After virus challenge, all lead VLP-immunized groups showed a significant 3- to 4-Log_10_ reduction in mean ZIKV RNAemia levels compared with control mice immunized only with alum, but the RNAemia reduction of 0.5 Log_10_ for F108A groups was statistically similar to the control. Successful viral control by the lead VLP candidate following challenge supports further vaccine development for this candidate. Notably, nAb titer levels in the lead, but not F108A, VLP-immunized mice inversely correlated with RNAemia. Further evaluation of sera by an *in vitro* Ab-dependent enhancement assay demonstrated that the F108A VLP-induced immune sera had a significantly higher capacity to promote ZIKV infection in FcγR-expressing cells. These data indicate that a single amino acid change in the fusion loop resulted in increased VLP yields but that the immature F108A particles were significantly diminished in their capacity to induce nAbs and provide protection against ZIKV challenge.

## Author summary

Zika virus (ZIKV) is transmitted by mosquitoes and is a serious health threat due to potential epidemic spread. Infection in adults may lead to Guillain-Barré syndrome, a neurological disorder, or may cause harm to a developing fetus resulting in preterm birth, fetal death, or devastating congenital malformations. There are currently no approved vaccines against ZIKV. We previously developed a lead candidate vaccine based on a virus-like particle (VLP) that was generated in tissue culture. This ZIKV shell is devoid of any viral genetic material. In previous studies, this lead VLP candidate generated neutralizing antibodies (nAbs) that recognized wild-type ZIKV and prevented viral replication in both mice and non-human primates. To increase production of the lead VLP candidate and decrease cost-of-goods, we introduced a single amino acid change, phenylalanine to alanine, in the envelope glycoprotein. This change resulted in a modest increase in VLP yield. However, this single amino acid change resulted in reduced induction of nAbs following immunization and no significant reduction of RNAemia following challenge compared to the lead candidate. The results of this study suggest this investigational vaccine candidate is not suitable for further vaccine development and that ZIKV VLP maturation may have an important role in protection.

## Introduction

Zika virus (ZIKV) is a mosquito-borne positive-sense RNA virus belonging to the *Flaviviridae* family, genus *Flavivirus*, which includes other disease pathogens such as dengue (DENV), West Nile (WNV), Tick-borne encephalitis (TBEV), yellow fever (YFV), and Japanese encephalitis (JEV) viruses [1]. ZIKV emerged in several major outbreaks first recorded in Yap Island, Micronesia in 2007, followed by French Polynesia in 2013, and subsequently Brazil and the Americas in 2015/2016 [2–4]. It was during the 2013 outbreak that Guillain-Barré syndrome was first described in association with ZIKV infection [5, 6]. Additional pathologies were observed during the ZIKV outbreak in Northeastern Brazil where evidence accumulated supporting a causal relationship between ZIKV infection of pregnant women and fetal injury [7–15]. In February 2016, the World Health Organization declared ZIKV disease a public health emergency of global concern.

During flavivirus replication, the viral genome encodes a polyprotein precursor that is processed into 3 structural proteins [capsid, pre-membrane/membrane (prM/M), envelope (E)] and 7 nonstructural proteins [16]. The pr peptide covers the tip of the E protein and prevents the viral particle from undergoing premature fusion within the secretory pathway of the host cell. The immature virion with trimeric spikes of prM-E dimers is cleaved by a host furin-like serine protease within the trans-Golgi to produce a mature ZIKV particle with a smooth outer shell made of 90 heterodimers of E and M proteins [17–19]. However, the prM cleavage by furin may be incomplete, and thus, a virion with both mature and immature regions are released from infected cells [1, 19–22]. The role that virion maturity has on protection is not completely understood and could have implications when developing vaccines [23, 24].

Although several candidates are in clinical development, currently there are no approved ZIKV vaccines that prevent viral infection of the mother and transmission to the developing fetus [25–34]. When developing vaccines against ZIKV, the potential for vaccine-induced disease enhancement is an important consideration. Specifically, a waning vaccine-induced antibody (Ab) response may increase disease severity following homotypic ZIKV infections or heterotypic flavivirus infections. In support of the latter concept, Katzelnick and colleagues demonstrated that ZIKV infection may enhance the future risk of severe dengue disease in humans [35]. Studies to address disease enhancement in previously ZIKV-infected populations may be possible to conduct when another outbreak occurs in a population with sufficient ZIKV seroprevalence. One potential mechanism of disease enhancement is known as antibody-dependent enhancement (ADE). ADE occurs when Ab-coated viruses bind to Fc gamma receptor (FcγR)-expressing cells (e.g., monocytes, macrophages, and dendritic cells) and enter these cells via FcγR-mediated endocytosis, leading to a significant increase in viral load and production of pro-inflammatory mediators [36–42]. With the goal of developing a safe ZIKV vaccine, the propensity of the ZIKV VLP vaccine candidates to induce ADE *in vitro* was evaluated herein. While direct demonstration of *in vivo* ADE in the mouse model may provide a more definitive answer as to the ADE-inducing potential of an early development vaccine candidate [43], measuring ADE *in vitro* can help with candidate down-selection and provide rationale for further development and evaluation in NHP studies that best align with observations in human populations [44]. ADE is most studied in dengue disease, whereby infection with one DENV serotype induces short-term heterologous protection against other DENV serotypes. However, protection wanes after a few months and infection with a different DENV serotype is more likely to promote dengue hemorrhagic fever or dengue shock syndrome [45–47]. A relevant case study is the licensed live-attenuated, chimeric yellow fever tetravalent dengue vaccine, which is currently approved in the USA only for individuals 9 through 16 years of age with laboratory-confirmed previous DENV infection [48, 49]. During phase III trials, this vaccine afforded 30% to 64% protection in vaccinees with previous DENV exposure, while in contrast, the number of hospitalizations and severe dengue disease cases increased in the DENV-naïve vaccine recipients [50–58].

Due to their ability to induce strong and durable Ab responses, virus-like particles (VLPs) are valuable platforms for vaccine development, as illustrated by licensed HPV vaccines [59]. Indeed, the organized, highly repetitive and dense manner in which VLPs display antigens on their surface enables the development of potent B-cell immunity [60]. Previously, we demonstrated that passive transfer of immune sera induced by the lead ZIKV VLP vaccine candidate protected AG129 mice against lethal ZIKV challenge [61]. In addition, a second study demonstrated that active immunization using the VLP vaccine also protected rhesus macaques against ZIKV challenge [62]. Herein, we sought to develop a ZIKV vaccine based on VLPs consisting of M and E structural proteins produced in mammalian cells to elicit protective neutralizing antibody (nAb) responses.

An important goal of the vaccine development program and the focus of this study was to increase the yield of ZIKV VLPs. A robust and scalable manufacturing process is necessary for a VLP platform to be cost-effective for widespread application and to compete with other vaccine candidates [63]. An earlier study by Urakami and colleagues evaluated DENV serotype 1 (DENV-1) VLP production of 27 different prM/M and E constructs and found that a phenylalanine to alanine amino acid change in position 108 (F108A) of the fusion loop (FL) promoted high VLP yields, approximately 16-fold above VLPs with wild-type prM/M and E amino acid sequences [64]. The mutation of key residues in E was predicted to induce conformational changes in DENV VLP and confer a VLP production advantage. Because the flavivirus FL in domain II of the E protein (EDII) is highly conserved [65–67], we hypothesized that the F108A mutation may also increase ZIKV VLP production, reduce the induction of non-neutralizing cross-reactive anti-E Abs, and maintain the induction of highly protective nAb responses.

We found that an F108A mutation in the ZIKV VLP construct resulted only in a modest increase in VLP yield and failed to induce protective nAbs in AG129 mice relative to the lead VLP vaccine candidate. Upon evaluation of the F108A VLP, it was observed that the particles were not processed to a mature state and that *in vitro* ZIKV infection was enhanced by F108A VLP-induced immune sera relative to the parent VLP particle. This lack of prM processing by furin could, in part, explain the lack of capacity to induce potent protective Ab responses.

## Materials and methods

### Ethical statements

#### Ethics statement for AG129 mice

Animal procedures were performed at University of California, Berkeley. AG129 mice were produced from in-house colonies and bred under specific pathogen-free conditions in an animal facility at the University of California, Berkeley. Veterinary care and experimental procedures were performed with the approval of the Institutional Animal Care and Use Committee. Mice were euthanized if weight loss was equal or greater than 20% of their original weight [61]. Study approval and animal care was conducted in accordance with the Guide for Care and Use of Laboratory Animals and U.S. Government Principles for the Utilization and Care of Vertebrate Animals Used in Testing, Research and Training.

### ZIKV VLP DNA design, production, purification, and characterization of VLPs

The ZIKV cassette plasmid construct used to generate the VLP has been previously described [61]. Briefly, the cassette containing the chimeric ZIKV structural genes, prM/M (African MR766 strain) and E (Brazilian SPH2015 strain), was inserted downstream from a human cytomegalovirus (CMV) IE enhancer/promoter in the CMV/R plasmid described by Barouch and colleagues [68]. The prM/M sequence encodes 168 amino acids (pr peptide = 93 amino acids and M peptide = 75 amino acids for stem and lipid membrane regions). The E sequence encodes 504 amino acids. The plasmid was electroporated into HEK293T cells, and supernatant containing ZIKV VLPs was subsequently harvested.

For VLP purification, the VLP-laden supernatant was collected 2–6 days post-transfection, clarified by centrifugation at 10,000×g for 10 min at room temperature, and pooled for VLP purification. Briefly, the supernatant was filtered through a diatomaceous Earth/Sartopore 2XLG column, concentrated 30× by Tangential Flow Filtration (TFF), and treated with 100U/mL benzonase for 1 hour. The supernatant was then buffer exchanged into 100mM sodium citrate dihydrate, 25mm Tris hydrochloride (pH 8.5); run through a CaptoCore 700 column; then buffer-exchanged into 300mM sodium citrate dihydrate, 25mm Tris hydrochloride (pH 8.5) prior to Size Exclusion Chromatography (SEC) on a Sephacryl 500 column. Pooled VLP-enriched fractions were concentrated on a 500-kDa hollow fiber column to ~1500x final volume in 25mm sodium citrate dihydrate, 25mm Tris hydrochloride (pH 8.5). The purified VLPs were sterile filtered prior to administration to animals. VLP concentration was quantified using a standard BCA assay (Thermo cat#23227).

For characterization, proteins from harvested supernatant or column-purified VLP samples were separated using NuPAGE 4–12% Bis-Tris precast protein gels (Invitrogen, CA) and either stained with InstantBlue Coomassie stain reagent (Abcam, England) or transferred onto a nitrocellulose membrane using an iBlot dry blotting system (Invitrogen, CA). Nitrocellulose membranes were blocked with 5% milk in phosphate buffered saline containing 0.05% Tween 20 (PBS-T). Rabbit anti-prM polyclonal Ab (Genetix, cat#gtx133305) and rabbit anti-ZIKV E Ab (MyBiosource, cat#mbs568050) at 1:1000 dilution were used to detect VLP components. After three PBS-T washes, membranes were probed with goat anti-rabbit HRP-conjugated Ab (Invitrogen, CA) at 1:10,000 dilution, and peroxidase-bound proteins were revealed using a SuperSignal West Femto kit (Thermo, cat#34094). The purified VLPs were further characterized by negative staining using uranyl acetate and visualized by transmission electron microscopy. Particles were predominantly found to be 30 and 55 nm in diameter.

### Biolayer Interferometry (BLI) Analysis of VLPs

VLPs were evaluated for their binding capacity against a panel of anti-ZIKV monoclonal Abs (mAbs), most of which are ZIKV-neutralizing. BLI measured the real-time mAb-VLP interactions using the Octet RED96 system (FortéBIO, Menlo Park, CA). Standard curves based on the lead ZIKV VLP were performed for each mAb to determine the relative binding of the F108A particle (S1 Fig). Specifically, lead VLP (100ng/mL to 3.125μg/mL) was bound to the indicated capture mAb, and wavelength changes (Δλ) were measured at 100 and 250 seconds (s). F108A VLP dilutions (25 to 6.25μg/mL) were bound under the same conditions by each mAb, and Δλ was measured. A 4PL regression curve of the lead VLP wavelength change to VLP concentration was generated using 250s binding time for mAbs with low affinity for F108A (ZIKV-117, ZV-48, and LM-081), while 100s was used for the others. The following mAbs were used: ZIKV-116, a nAb that blocks ZIKV fusion with host membranes and recognizes the lateral ridge of E Domain III (EDIII) [69]; ZIKV-117, a nAb with broad activity against African and Asian lineages that binds to a quaternary interdimer epitope on E [70–72]; ZIKV-67, a nAb that binds to the lateral ridge of EDIII [73]; ZIKV-48, a nAb that binds the C-C loop of EDIII [73]; IM-79, a non-neutralizing mAb that binds to ZIKV reporter virus particles (Integral Molecular, Philadelphia, PA); and LM-081, a nAb that binds to quaternary epitopes in EDII and EDIII of adjacent ZIKV E protein monomers [74].

### ZIKV challenge strain

ZIKV clinical isolate Nica 2–16 was originally isolated from a ZIKV-infected Nicaraguan patient in 2016 by the National Virology Laboratory, Ministry of Health, Managua, Nicaragua [75]. The virus was propagated in C6/36 cells to generate the virus stock and titered by a focus-forming assay on BHK cells [17].

### Immunogenicity and efficacy studies

AG129 mice used in this study lack the receptor for types I and II IFN (IFN α/β and γ) and display age-dependent morbidity and mortality following ZIKV infection, providing a platform for testing the efficacy of antivirals and vaccines. Eleven- to sixteen-week-old male and female AG129 mice were immunized (n = 5 mice per group) with ZIKV lead VLP or F108A VLP (50μL each hind limb, 100μL total) by the intramuscular (IM) route. Animals were immunized on Day 0 followed by a booster immunization given on Day 28 with doses of 0.1, 1, or 10μg of VLP in combination with 100μg Alhydrogel 2% (alum) adjuvant (Brenntag Biosector A/S, cat# vac-alu-250). Control groups consisted of mice immunized with 100μg of alum (Alum-only, no VLP) as well as of unimmunized mice (Naïve). In addition, control mice that were unimmunized and not challenged with ZIKV (Uninfected) were also included in these experiments. Male and female mice were distributed as evenly as possible in groups to avoid a potential sex bias. Blood samples were collected 21 days after each immunization (Day 21 and Day 49) to evaluate induction of ZIKV-specific Abs. Mice were challenged 52 days after the initial immunization (24 days following booster immunization) with 5,000 focus-forming units (FFU) of ZIKV clinical isolate Nica 2–16 delivered subcutaneously (SC) in the left hind footpad in a 50μL volume. Blood specimens were collected on days 2 and 4 post-ZIKV challenge (Days 54 and 56 after the first immunization) to quantify ZIKV relative genomic RNA copies by RT-qPCR (GE/mL). Additionally, mice were weighed daily through 20 days post-virus inoculation.

### ZIKV-specific assays

#### VLP-capture ELISA

Total serum IgG levels to Zika VLP were measured by sandwich ELISA using VLP as the capture antigen. To optimize signal-to-noise ratio, capture Ab, VLP antigen, and secondary Ab concentrations were defined using a positive control serum sample (pooled Day 21 sera from mice immunized with 1 or 10μg dose of F108A VLP). Flat-bottom 384-well plates were coated overnight at 4°C with rabbit serum containing Abs raised against prM and E and diluted 1:4,000 in carbonate-bicarbonate buffer (Sigma, cat#C3041). The next day, plates were blocked with 5% nonfat dry milk in PBS-T, and 40ng of VLPs in blocking buffer were added to each well and incubated for 2 hours at 37°C. Murine immune serum samples were serially diluted 1:50 to 1: 21,870, and each dilution was tested in duplicate. Overnight incubation with 20μL test sera at 4°C was followed by detection of binding Abs with 7.5ng/well goat anti-mouse IgG conjugated to horseradish peroxidase (Invitrogen, cat#626520) in blocking buffer for 1 hour at 37°C. TMB substrate was added and incubated for 10 min, with stop solution (KPL, cat#50-85-05) being added prior to the measurement of optical density (OD) at a wavelength of 450nm (OD_450_). Endpoint titers were defined as the reciprocal dilution that resulted in an OD_450_ = 0.1 using 4PL regression and GraphPad Prism 8 analysis software. Sera with undetectable binding were assigned a value one-half LOD (25). The concentrations of lead and purified F108A particles used as capture antigens was determined prior to ELISA by BCA assay. The positive control mouse anti-ZIKV E serum (zoonogen, cat#pa10049) was diluted 1:3,200 to 1:21,870.

#### ZIKV *Renilla* luciferase neutralization assay (ZIKV-RlucNT_50_)

ZIKV-specific nAb titers in serum samples collected at the designated time points were determined using a luciferase-based virus neutralization assay as described by Espinosa and colleagues [61]. The assay is based on the capacity of Abs to neutralize recombinant ZIKV reporter virus generated from modified SPH2015 strain (ZIKV-Rluc; Integral Molecular, Philadelphia, PA), with readout consisting of inhibition of luciferase transgene expression. Neutralization (NT_50_) titers were defined as the reciprocal serum dilution that neutralizes 50% of luciferase activity using linear regression analysis. Serum samples with undetectable neutralization activity were assigned a value one-half LOD (5) for graphing and statistical analysis.

#### Quantitative Reverse Transcriptase PCR, AG129 mice

ZIKV RNA copy number was determined from 200μL of serum from blood samples collected via submandibular bleed on days 2 and 4 post-challenge. Blood was allowed to clot at room temperature, and serum was separated by centrifugation. ZIKV RNA was extracted from serum samples using the QIAamp Viral RNA Mini Kit (Qiagen), and RNA levels were determined by TaqMan one-step quantitative reverse transcriptase PCR (RT-qPCR) using a standard curve comprising 10-fold dilutions of ZIKV Nica 2–16 RNA in the range of 10^1^ to 10^8^ genome equivalents per mL (GE/mL) [61]. The RT-qPCR assay had no established LOD or LLOQ so all values of genome equivalents per mL serum (GE/mL) are presented and an arbitrary log LOD of -0.229 was set based on the lowest log GE/mL detected (0.59 GE/mL). Serum samples with undetectable ZIKV RNA (C_T_ ≥ 40) were assigned a log value of -0.53 (the log value of one-half the LOD) for graphing and statistical analysis.

#### Antibody-dependent enhancement assay (ADE)

FcγR-expressing K562-r cell line (ATCC, cat# CRL-3344) was cultivated in RPMI medium (2mM Glutamine, 10% FBS, 50μg/mL non-essential amino acids, 50μg/ml Gentamicin, and 10mM HEPES). Serum samples from VLP-immunized mice were serially diluted in duplicate from 1:10 to 1:2,430 with complete RPMI medium, mixed 1:1 (v:v) with ZIKV strain PRVABC59 (provided by the Centers for Disease Control and Prevention), and incubated for 1 hour at 37°C (targeted virus range for media only control: 10–12% of cells infected). Cells were added at 5 × 10^4^ per well to virus-serum complexes and incubated for 2 hours at 37°C. Cells were centrifuged, washed once with medium, resuspended in complete RPMI medium, and incubated at 37°C and 5% CO_2_ for 24 hours. On the day of staining, cells were collected by centrifugation, washed once with RPMI, and fixed with 5% formaldehyde for 10 min at room temperature. Alexa Fluor 488-conjugated 4G2 mAb, pan-specific against the flavivirus E protein, was diluted 1:500 in perm/stain buffer (0.1% Saponin and 2.5% BSA in PBS) and added to the cells for 30 min at room temperature. Cells were washed twice and resuspended in FACS buffer (0.5% BSA in PBS). Infection enhancement was calculated by dividing the mean percentage of ZIKV-infected cells treated with virus serocomplex by the mean percentage of cells infected with ZIKV in the absence of serum (i.e., in the presence of tissue culture medium only). The threshold for ADE activity was calculated by averaging the fold-enhancement values of all samples from the control groups (n = 15; Alum-only, Naïve and Uninfected groups) and adding 3 standard deviations (SD), similarly to previously described [64].

### Statistical analysis

GraphPad Prism 8.0.0 was used to plot results and for statistical analyses. The statistical analyses used for each dataset are described in each figure legend. Where noted, assay results below the detection limit were assigned a value one half the detection limit for graphing and statistical analysis. ELISA titers in the VLP and Alum-only groups were compared by one-way ANOVA. Lead VLP/F108A VLP relative binding ratios were compared by one-way ANOVA followed by Sidak’s multiple comparison tests between the lead VLP and F108A VLP groups at each dose level. Combined lead VLP-binding ratios were compared to those of the combined F108A data by unpaired t test. Serum nAb titers generated by RlucNT_50_ assay, NT_50_ titers normalized to endpoint ELISA titers, and RNAemia levels by RT-qPCR (GE/mL) were compared within each time point by Kruskal-Wallis nonparametric ANOVA followed by Dunn’s pairwise comparison tests between 1) the Alum-only control group and each VLP-immunized group (n = 6 comparisons) and 2) the lead VLP group and the F108A group at each dose level (n = 3 comparisons). Percentage weight changes in mice post-ZIKV challenge were compared on each day by one-way ANOVA followed by Dunnett’s multiple comparison tests with the Alum-only control group (n = 8 comparisons). Day 54 (day 2 post-challenge) log_10_ GE/mL values and Day 49 (3 weeks post-booster) RlucNT_50_ levels in the surviving versus dead mice were compared by Mann-Whitney U tests. Spearman correlation analysis of Day 49 RlucNT_50_ titers compared to Day 54 RNA GE/mL values were performed on log_10_-transformed values r and P values shown in the figure. Fold-enhancement of infection values were compared by one-way ANOVA followed by 1) Dunnett’s multiple comparison tests between the Alum-only control group and each VLP-immunized group (n = 6 comparisons) and 2) Sidak’s pairwise comparison tests between the lead VLP group and the F108A group at each dose level (n = 3 comparisons). All P values are 2-tailed, and significance levels are denoted as: ****P < 0.0001, ***P < 0.001, **P < 0.01, *P < 0.05, or ns (not significant).

## Results

### A single amino acid change at position 108 in the E protein fusion loop of ZIKV VLP increases particle production approximately 2-fold with no apparent change in particle morphology

**Fig 1:**
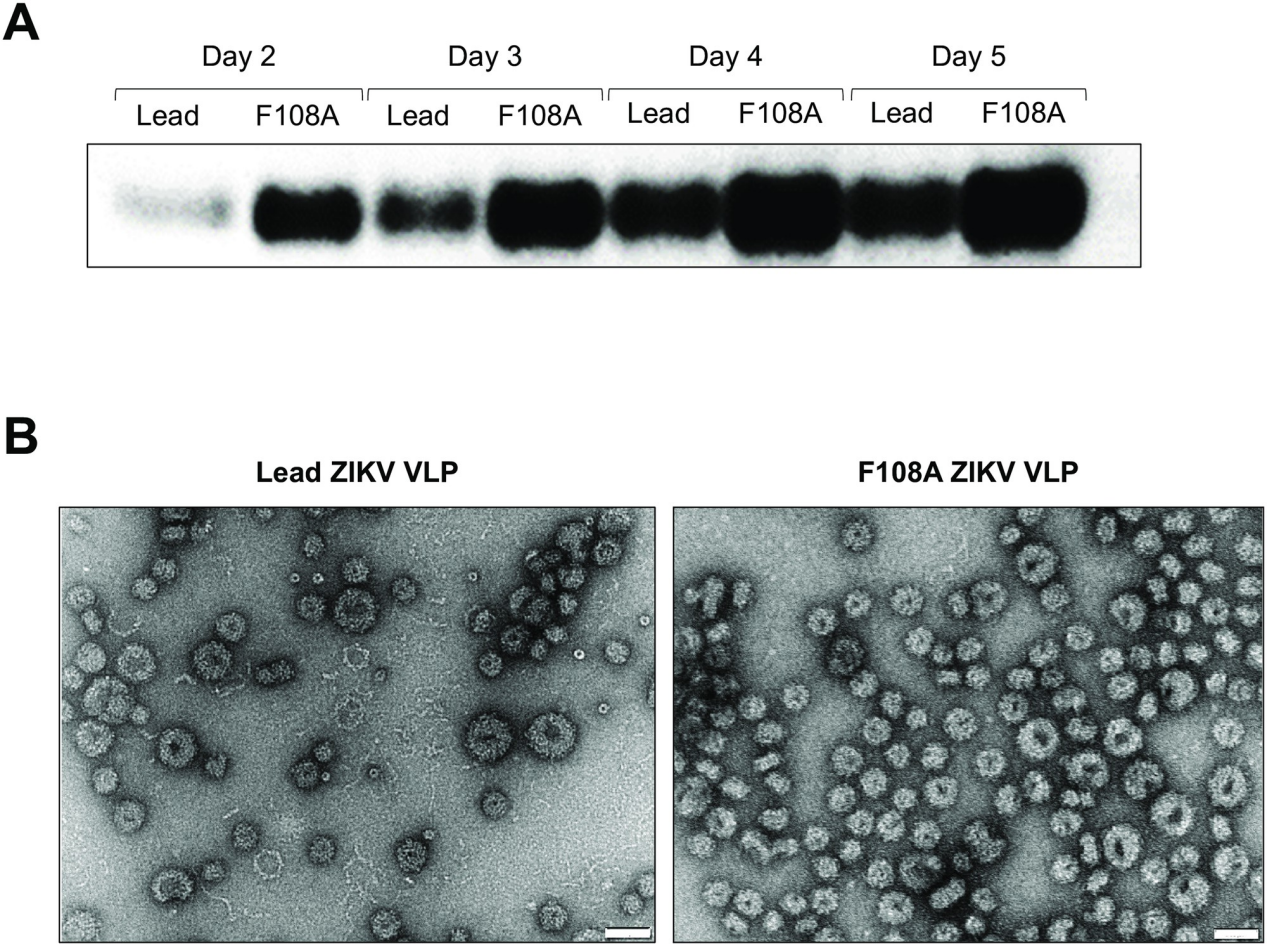
Lead and F108A ZIKV VLP production and Transmission Electron Microscopy (TEM). (A) Western blot of supernatant samples collected on days 2, 3, 4, and 5 days post-transfection (p.t.) from cells transfected with a plasmid containing either the lead or F108A ZIKV VLP cassette. Proteins were stained for E using a mouse ZIKV-specific mAb (MyBiosource, cat#mbs568050). (B) Transmission electron micrographs of purified lead (left panel) and F108A VLPs (right panel) negatively stained with uranyl acetate. The white scale bar depicted on images is 50nm long.

The goal of this study was to test whether a mutation could be introduced into the E protein of a lead ZIKV VLP vaccine candidate in order to confer an increase in yield without negatively affecting VLP immunogenicity and efficacy. A single point mutation introduced into the nucleotide sequence of the E protein was sufficient to induce an amino acid change from a phenylalanine to an alanine at position 108 (F108A). To characterize the mutated VLP vaccine candidate F108A, we transfected HEK293T cells with the F108A VLP and the ZIKV lead vaccine candidates in parallel under the same growth conditions. Analyzing the E protein content of clarified supernatant harvests revealed a modest yield increase of extracellular E protein detected in the F108A preparation compared to that of the lead VLP harvest. Increased VLP yields of F108A were evident by Western blot analysis on days 2, 3, 4 and 5 post-transfections (Fig 1A). Additionally, in 3 of the development runs of VLP upstream production, VLP capture ELISA quantification showed apparent ca. 2-fold yield increases for F108A that corroborated the Western blot data. Intact spherical particles with a diameter of approximately 30nm and 50nm were found in both lead and F108A VLP preparations (electron microscopy, Fig 1B). No other morphological distinctions between ZIKV lead and F108A VLPs based on the electron microscopy could be ascertained at this resolution. Together, these results demonstrated a modest yield benefit for the F108 mutant without apparent changes to VLP morphology.

### The F108A mutation in the E protein FL affects processing of the prM protein and VLP epitope availability

**Fig 2:**
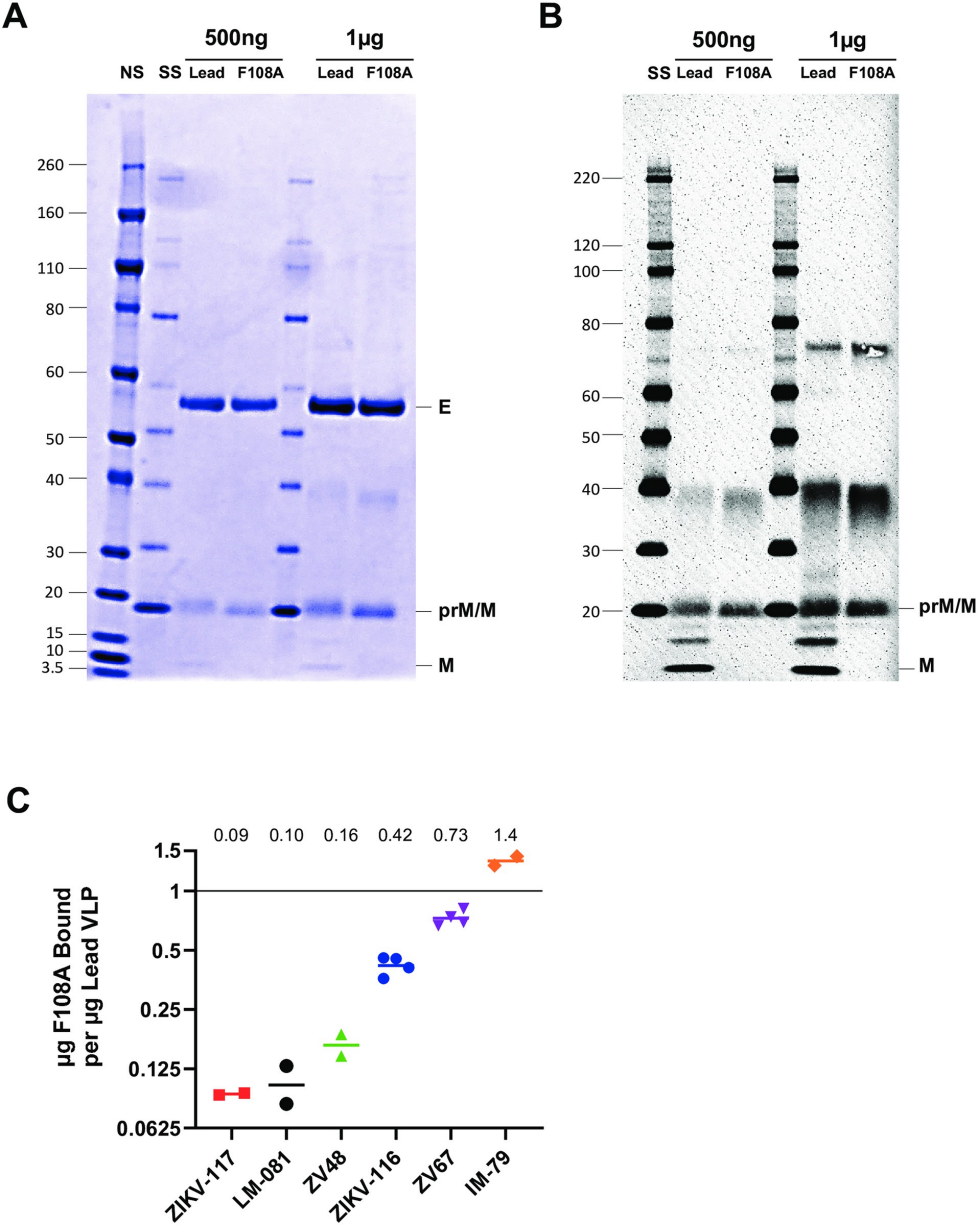
Comparison of the maturation state of lead and F108A particles by prM/M protein detection and E epitope mapping. (A) SDS-PAGE followed by Coomassie protein staining of 500ng and 1μg of purified lead and F108A ZIKV VLPs. Molecular weight ladders on this gel are the Novex Sharp (NS; Invitrogen, cat#57318, molecular weights for this ladder shown in kDa) and the Super Signal (SS; Thermo, cat#847868) (B) SDS-PAGE followed by immunoblotting analysis of 500ng and 1μg of purified lead and F108A ZIKV VLPs. An anti-prM polyclonal Ab against ZIKV (Genetex, cat#gtx133305) was used to detect VLP proteins. (C) Binding of each mAb to F108A VLP relative to lead VLP measured by Biolayer Interferometry. Binding was measured at 100 seconds (ZIKV-116, ZV67, IM-79), 250 seconds (ZIKV-117, ZV48, LM-081) or both 100 and 250 seconds (ZIKV-117). For ZIKV-117, only the highest F108A concentration tested fell on the standard curves for the 100 and 250-second measurements, so both are shown. For each Ab, 2 or 4 F108A VLP dilutions fell on the lead VLP standard curve. For those dilutions, the F108A VLP concentration was calculated by least squares fit from the 4PL curve, and the ratios of bound F108A VLP to bound lead VLP were calculated. Refer to S1 Fig for standard curves. Symbols represent the ratios from individual measurements, bars and numbers at the top of each group represent geometric mean ratios, and an equivalence line representing equal mAb binding to F108A and lead is shown at 1.

Particle maturation occurs upon cleavage of prM by host cell furin, which promotes the rearrangement of the E proteins into dimers that lie flat along the virion membrane. PrM cleavage in purified ZIKV VLPs was analyzed by SDS-PAGE with Coomassie Blue staining and Western blot. Purified lead and F108A VLP preparations were run side-by-side, and contaminants in both preparations were observed to be minimal by Coomassie staining in the 1μg lane, the higher mass tested (Fig 2A). The E protein band (54kDa) and the prM/M band (18.5kDa) intensities remained comparable between lead and F108A VLPs. M protein (ecto-domain + transmembrane domain) at 8kDa was only detected in the ZIKV VLP lead preparation, suggesting that the F108A mutation prevented detectable cleavage of prM into its smaller components pr (10kDa) and M (8kDa) (Fig 2A and 2B). Data presented in S2 Fig illustrating the evaluation of a different lot of ZIKV F108A and lead VLPs support these observations. Column-derived F108A and lead VLP pooled fractions (S2A and S2B Fig, respectively) when evaluated by Western blot (S2C Fig) indicate a visible band corresponding to M protein identified with the lead ZIKV VLP but not the F108A VLP preparation. The identity of the 15kDa band in Fig 2A and 2B is unknown but potentially a degradation product of a precursor protein. The absence of pr at 10kDa may be due to a loss of the cleaved peptide during purification. Analysis of the prM processing provided insight into the level of particle maturation in both preparations. We observed that the ZIKV VLP lead particles were partially mature or mixed whereas F108A particles lacked detectable maturation and thus were relatively immature. Absence of F108A particle maturation may have led to the modest increase of the VLP yield.

To provide further characterization of the F108A VLP structure, BLI was used to evaluate the availability of known epitopes of E on F108A particles relative to lead particles using a panel of previously characterized mAbs specific for ZIKV (Fig 2C). Five neutralizing and one non-neutralizing mAbs were evaluated by BLI for binding to lead and F108A particles. Standard curves for each mAb were created based on known VLP concentration and used to quantify the relative F108A VLP binding (S1A Fig). Binding ratios of F108A VLP relative to lead VLP were calculated for each F108A concentration that fell on the lead standard curve for that mAb, and these ratios are shown for each mAb in Fig 2C. We found that relative to the lead VLP, binding of F108A VLP was reduced using all neutralizing ZIKV mAbs, with average binding ratios from 0.73 to 0.09. Ab binding to F108A VLP was found to be the most reduced relative to lead VLP by mAb ZIKV-117, a neutralizing mAb that binds to a quaternary interdimer epitope on the E dimer-dimer interface on EDII, the same domain as F108A. For ZIKV-117, binding was reduced by 11-fold (binding ratio of 0.093) relative to lead. Neutralizing mAb LM-081, which binds to quaternary epitopes in EDII and EDIII, showed similarly reduced binding to F108A that was 10-fold reduced relative to lead (binding ratio of 0.10). ZV-48 binds the C-C loop of EDIII and showed 6-fold reduced binding of F108A. By contrast, ZIKV-116 and ZV-67, which both bind to the lateral ridge of EDIII showed comparable binding to F108A and lead VLP, with binding ratios of 0.42 and 0.73, respectively. Finally, IM-79, which was found to bind to ZIKV-Rluc particles but without detectable neutralizing activity (product technical data, Integral Molecular, Philadelphia, PA), showed a modest increased binding to F108A relative to lead VLP, demonstrating that not all mAbs binding to F108A were reduced. Thus, these VLP characterization data together provide evidence of the relative immaturity of the F108A VLP compared to the lead and suggested ultrastructural differences in at least some of the neutralizing epitopes of E.

### VLP binding Ab responses are comparable after immunization with lead or F108A VLPs

**Fig 3:**
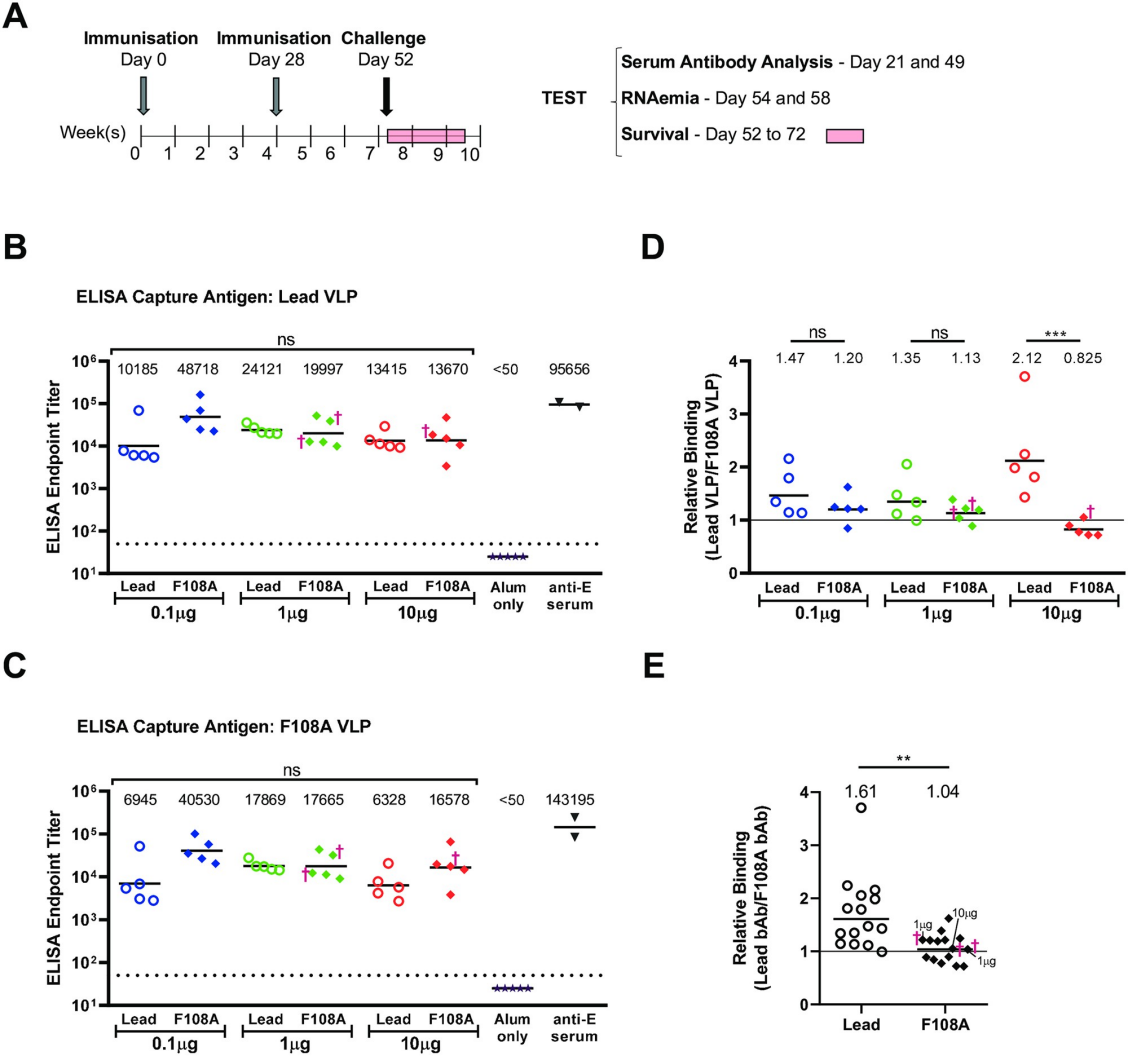
Vaccination and induction of lead and F108A VLP binding Abs. (A) Vaccination and bleeding schedule of AG129 mice are shown. Mice (5 per group) were immunized IM on day 0 and day 28 with doses of 0.1, 1, or 10μg VLP and alum adjuvant. Mice were subsequently SC challenged with ZIKV (Nica 2–16 strain) on day 52. Bleeding was performed on Days 21 and 49 of the study prior to viral challenge. Day 49 sera were tested by sandwich ELISA using captured lead (B) or F108A (C) VLPs, and endpoint titers were calculated. Each symbol represents the mean endpoint dilution of two independent measurements for an individual animal, and the geometric mean titer is depicted by a horizontal bar. The dotted line is the limit of detection (LOD) of the assay, and sera with undetectable binding were assigned a value of one-half LOD (25). Anti-E ZIKV mouse serum (Zoonogen, cat#pa10049) was used as a positive control and to demonstrate binding availability of E for the VLP types. For each capture antigen, endpoint titers of the VLP groups were compared by one-way ANOVA. The symbol † denotes a mouse that succumbed after viral challenge. (D) Relative Binding is the ratio of the lead VLP ELISA endpoint titer divided by the F108A VLP ELISA endpoint titer for each individual sample. Group geometric mean ratios are shown on top, and an equivalence line was placed at y = 1. One-way ANOVA followed by Sidak’s multiple comparison tests for each of the three doses were performed. (E) Comparison of Relative Binding Antibody (bAb) for combined lead vs combined F108A VLP groups is shown. Combined group geometric mean ratios and significance level by unpaired t test are shown on top. Dose group of F108A VLP-immunized mice that succumbed later to viral challenge is indicated. Comparison of the combined groups was by Mann-Whitney U test (ns, not significant, *P<0.05, **P<0.01, ***P<0.001, ****P<0.0001).

We next tested the immunogenicity and protection resulting from immunization with the two VLP types which had apparent differences in particle maturity. AG129 mice were immunized twice, one month apart (Days 0 and 28) with a dose titration of 0.1, 1, or 10μg of either of the two VLP candidates together with 100μg alum adjuvant. Virus challenge was on Day 52, approximately 1 month following the booster immunization, as shown in the study schedule (Fig 3A). To quantify the levels of ZIKV VLP-specific binding IgG in the mice after the booster immunization, sandwich ELISAs using the lead or mutant VLPs as the capture antigens were performed. We found that after two successive immunizations with the VLP candidates, endpoint titers of VLP-binding IgG were in the range of 6,000 to 49,000 against lead (Fig 3B) and F108A (Fig 3C) VLPs, but that titers were similar in all immunized groups regardless of VLP type or dose (ELISA titer comparisons of the 6 VLP immunized groups by one-way ANOVA; Lead, *P* = 0.105, F108A, *P* = 0.068). As expected, Ab titers in alum-only immunized mice were below the limit of detection while the positive control, anti-E mouse serum, resulted in Ab titers of approximately 96,000 and 143,000 for lead and F108A VLP antigens, respectively.

Because of the apparent differences in VLP maturation and epitope abundance between the two VLP types used for immunization, we asked whether the IgG specificities raised in each mouse had a detectable preferential binding to one of the two VLP types. To calculate the relative binding for each serum, the ELISA IgG titer for the lead VLP particle was divided by the F108A VLP titer. We found that at the 10μg VLP dose, the serum IgG from the lead VLP immunized mice showed a significant 2.6-fold increased binding to lead VLP relative to F108A (*P* = 0.0002, Fig 3D). Specifically, while the IgG to F018A showed slightly higher binding to F108A VLP (relative binding geometric mean of 0.825), IgG to lead VLP showed a geometric mean 2.12-fold higher binding to lead VLP. The two other dose groups showed only slightly higher binding ratios in the lead VLP groups compared to F108A groups (*P*>0.05 for these doses). Because no apparent dose response was evident based on the ELISA IgG titers comparing each of the 3 VLP groups, we examined the overall effect on binding ratios for the combined lead VLP groups compared to the combined F108A VLP groups. Data presented in Fig 3E show that, overall, the IgG of the combined F108A groups bound lead and F108A VLPs comparably (geometric mean binding ratio of 1.04). However, the combined lead VLP groups showed a significantly increased binding (*P* = 0.002) to lead VLP (geometric mean binding ratio of 1.61). Taken together, these results show that the IgG raised to lead VLP exhibited higher binding to lead VLP compared to F108A VLP and that significant binding preferences toward lead and F108A VLPs were evident only at the highest VLP dose groups. Thus, this suggests that F108A mutation-mediated effects on neutralization or protection against ZIKV may be most readily apparent at the 10μg dose.

### The nAb response against ZIKV in mice immunized with the lead VLP is greater than that of F108A mutant VLP

**Fig 4:**
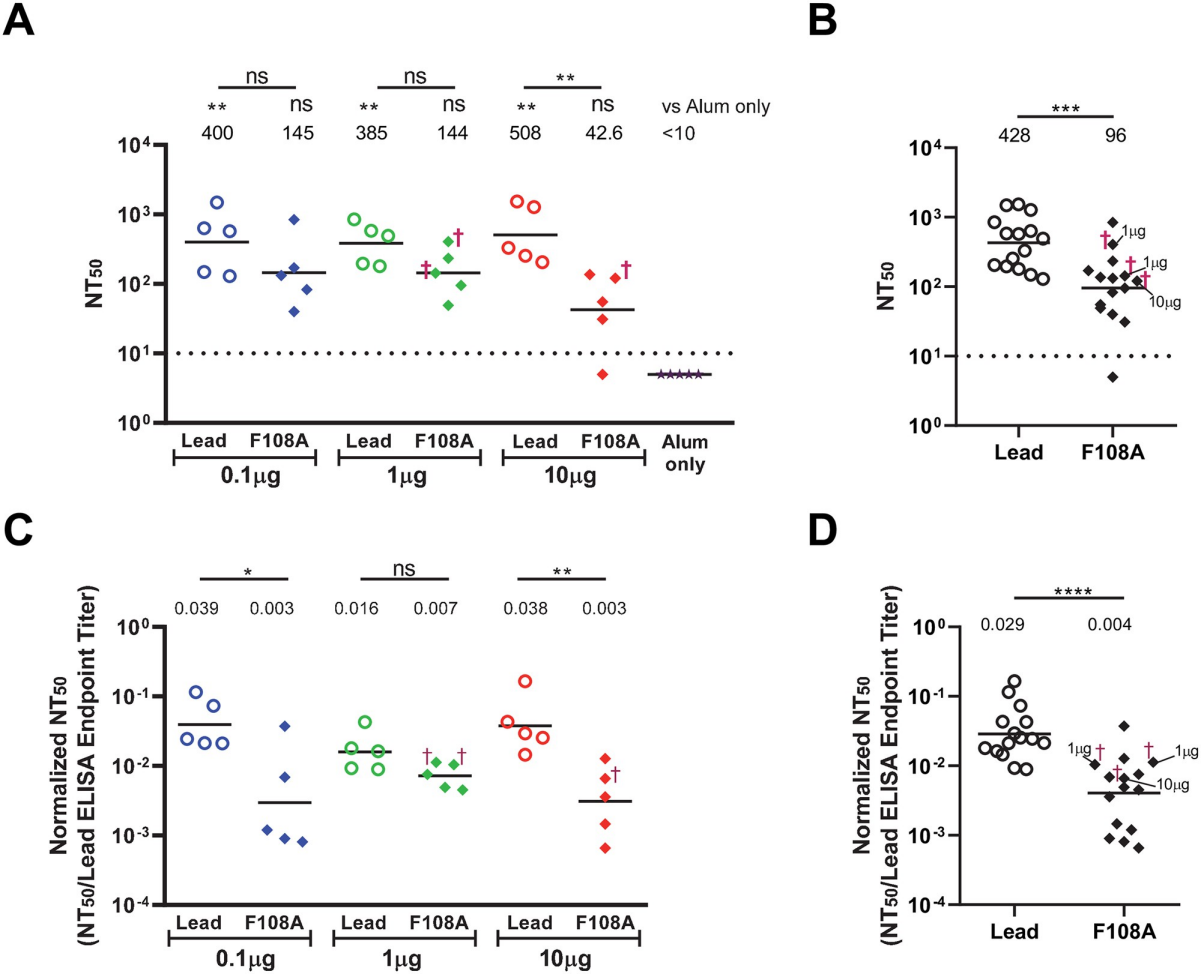
Lead and F108A VLP induction of nAb after booster immunization. The vaccination schedule is shown in Fig 3A. (A) NT_50_ is defined as the reciprocal dilution of serum that neutralizes 50% of reporter virus luminescence calculated using linear regression analysis. Each symbol represents the NT_50_ for an individual animal, and † indicates a lethal ZIKV infection. Horizontal bars represent the geometric mean titer (GMT) of each group. The dotted line is the limit of detection (LOD) of the assay, and sera with undetectable neutralization activity were assigned a value of one-half LOD (5). Statistical comparisons of NT_50_ data were performed using Kruskal-Wallis test followed by Dunn’s multiple comparisons tests (*P<0.05; **P<0.01; ***P<0.001; ****P<0.0001; ns, not significant). All VLP groups were compared to the Alum-only group. Additional pairwise comparisons were performed between titers of lead and F108A groups at each dose, with significance level for each comparison shown above each bar. (B) Comparison of NT_50_ titers for combined lead VLP groups compared to combined F108A VLP groups. GMT, individual NT_50_ values, assay LOD, † designation, and significance value by Mann-Whitney U test are as in (A). (C) Ab quality assessment by the ratio of NT_50_ to ELISA titer to lead VLP are shown for each dose of lead or F108A VLP. Group geometric mean titer ratios are shown above each group, individual ratios are shown by a symbol, group means by a horizontal bar, lethal infections by the symbol †, and statistical significance by Kruskal-Wallis followed by Dunn’s multiple comparison tests for each of the three doses as in (A). (D) Ab quality assessment by NT_50_ to ELISA titer to lead VLP ratios are shown for combined lead and combined F108A groups. Group GMT ratios, individual ratios, lethal infections, and statistical significance levels by Mann-Whitney U test are as in (A).

Lead and F108A VLP-induced immune sera from AG129 mice were tested for their ability to neutralize ZIKV reporter particles from the Brazilian epidemic strain SPH2015 [74]. Of note, standard production of these reporter particles (in the absence of furin overexpression) was shown by the vendor to yield partially mature particles based on partial prM cleavage observed on Western blot. For sera collected on Day 21 following prime immunization, only the 1μg and 10μg lead VLP groups displayed significant nAb levels compared with the Alum-only control group (P = 0.0093 and 0.0006, respectively) (S3 Fig). Additionally, nAb titers were significantly higher in the 10μg lead VLP group compared to the 10μg F108A VLP group (P = 0.002, S3 Fig). The geometric mean nAb titers of the groups after the boost (Day 49), in the range of 40 to 510, are illustrated in Fig 4A. While the negative control (alum-only) mice showed no detectable neutralizing activity, 29 of 30 mice immunized with the ZIKV VLP constructs showed serum nAb activity; the only immune serum with NT_50_ <10 being observed in the 10μg F108A group (Fig 4A). Only the lead VLP groups showed GMTs that were significantly increased compared to the Alum-only control group (*P* = 0.0059, *P* = 0.0034, *P* = 0.0010 for the 0.1, 1, and 10μg doses, respectively) (Fig 4A). Similar to the binding Ab responses, the ZIKV reporter particle-specific nAb responses induced by the F108A VLP trended lower compared to the lead VLP following immunizations with 0.1μg (2.8-fold) and 1μg (2.7-fold) of VLP. A significant difference between NT_50_ titers was only observed in mice immunized with 10μg VLP (*P* = 0.0053), with F108A VLP inducing 11.9-fold less nAbs that the lead VLP (Fig 4A). A 4.5-fold overall decrease in NT_50_ titers was observed between combined lead and combined F108A VLP immunized groups (Fig 4B, *P* = 0.0002).

We next assessed the nAb response as a proportion of the total VLP-specific IgG response in each mouse as a measure of the quality of the immune response. To this end, we normalized each NT_50_ titer to its corresponding endpoint ELISA IgG titer against the lead VLP. We found that in mice immunized with 10μg of VLP, normalized NT_50_ titers were significantly 12.2-fold higher in the lead VLP immunized group compared to the Fl08A group (*P* = 0.008, Fig 4C). Normalized NT_50_ values in the 0.1μg dose groups were similar to those in the 10μg dose group, with a 13-fold lower antibody quality in F108A VLP-immunized mice (*P* = 0.019), while Ab quality values in the 1μg dose groups were similar (*P*>0.05). We observed an overall 7-fold increase in Ab quality in the combined lead groups compared to the combined F108A VLP groups as well (Fig 4D, *P*<0.0001). These nAb data demonstrated a significant difference in functional Ab levels elicited by lead or F108A VLPs and that the lead VLP generated a higher proportion of neutralizing IgG compared to the F108A particle.

### F108A VLP confers diminished protection compared to lead VLP against ZIKV challenge

**Fig 5:**
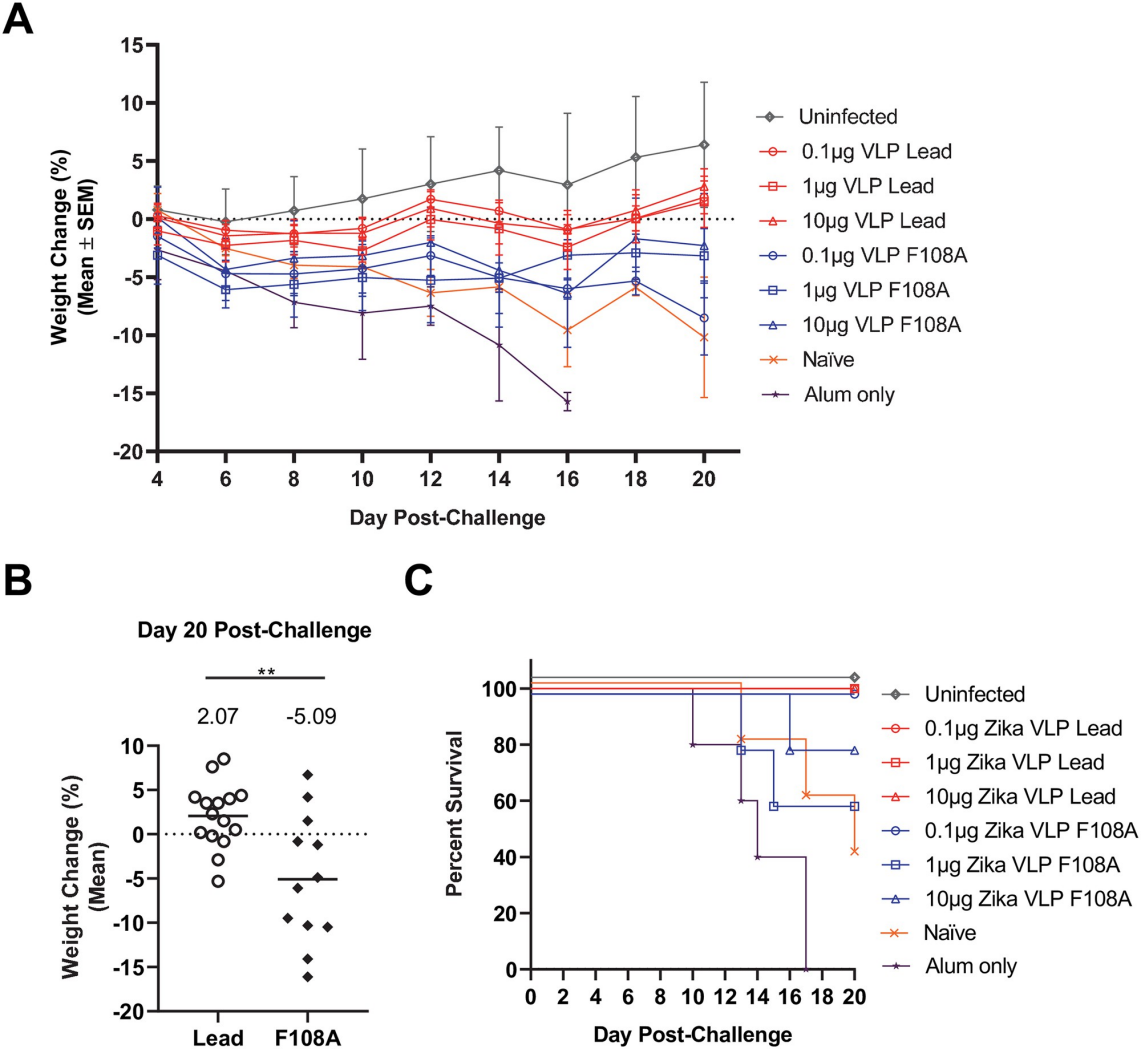
Protection afforded by VLP immunization as measured by weight loss and survival. The vaccination schedule is shown in Fig 3A. (A) Weight changes from day 4 post-challenge of F108A VLP-immunized (blue), lead VLP-immunized (red), and control [naïve challenged (naïve), alum-only immunized and challenged (alum-only), and naïve unchallenged (uninfected)] mice. Each symbol corresponds to the mean cumulative weight change on days 4 to 20 for each mouse group (n = 5), and error bars indicate standard error of the mean (SEM). Statistical comparisons of weight change per day were performed by one-way ANOVA followed by Dunnett’s multiple comparisons tests between all groups each day. Significance levels between control and VLP-immunized groups relative to the Alum-only group are indicated in S4 Fig. (B) Day 20 post-challenge weight changes were combined for F108A and lead VLP groups, respectively. Statistical significances were obtained by performing an unpaired t-test and means for each combined VLP dose groups are indicated on top. (C) Survival of mice was assessed up to 20 days post-challenge.

The VLP immunized mice were challenged SC with ZIKV strain Nica 2–16 and efficacy was evaluated by monitoring weight loss, RNAemia, and survival for 20 days after challenge. Beginning on day 4 post-challenge, weight loss of ZIKV challenged mice was compared to uninfected controls (Figs 5A and S4). Significant weight loss began to be evident for alum-only control mice compared to uninfected mice 8 days post-challenge (*P* < 0.05 on days 8, 10 and 12 and *P*< 0.01 on days 14 and 16). The Naïve group also showed significant weight loss relative to the Uninfected group beginning at day 12 through the end of the observation period on day 20 (*P* = 0.03 to 0.004). Importantly, on day 16 post-challenge, only the three lead VLP-immunized groups exhibited significantly higher weight relative to the Alum-only group (*P*<0.05 for each group), and by day 18, there were too few survivors in the Alum-only group to allow for continued comparisons (S4 Fig). At the end of the observation period, the lead VLP-immunized mice had significantly higher body weights compared to mice immunized with the F108A VLPs (Fig 5B). Survival upon ZIKV challenge was also assessed throughout the 20-day monitoring period (Fig 5C). Interestingly, all mice from the Alum-only group (n = 5) succumbed to infection by day 17, while only 3 out of 5 naïve mice died. Mice immunized with the lead VLP at all doses were fully protected. In contrast, only the F108A VLP at the 0.1μg dose afforded full protection while the 10 and 1μg F108A VLP doses protected 80% (4 out of 5) and 60% (3 out of 5) animals, respectively.

### F108A VLP confers diminished protection compared to lead VLP against RNAemia following virus challenge

**Fig 6:**
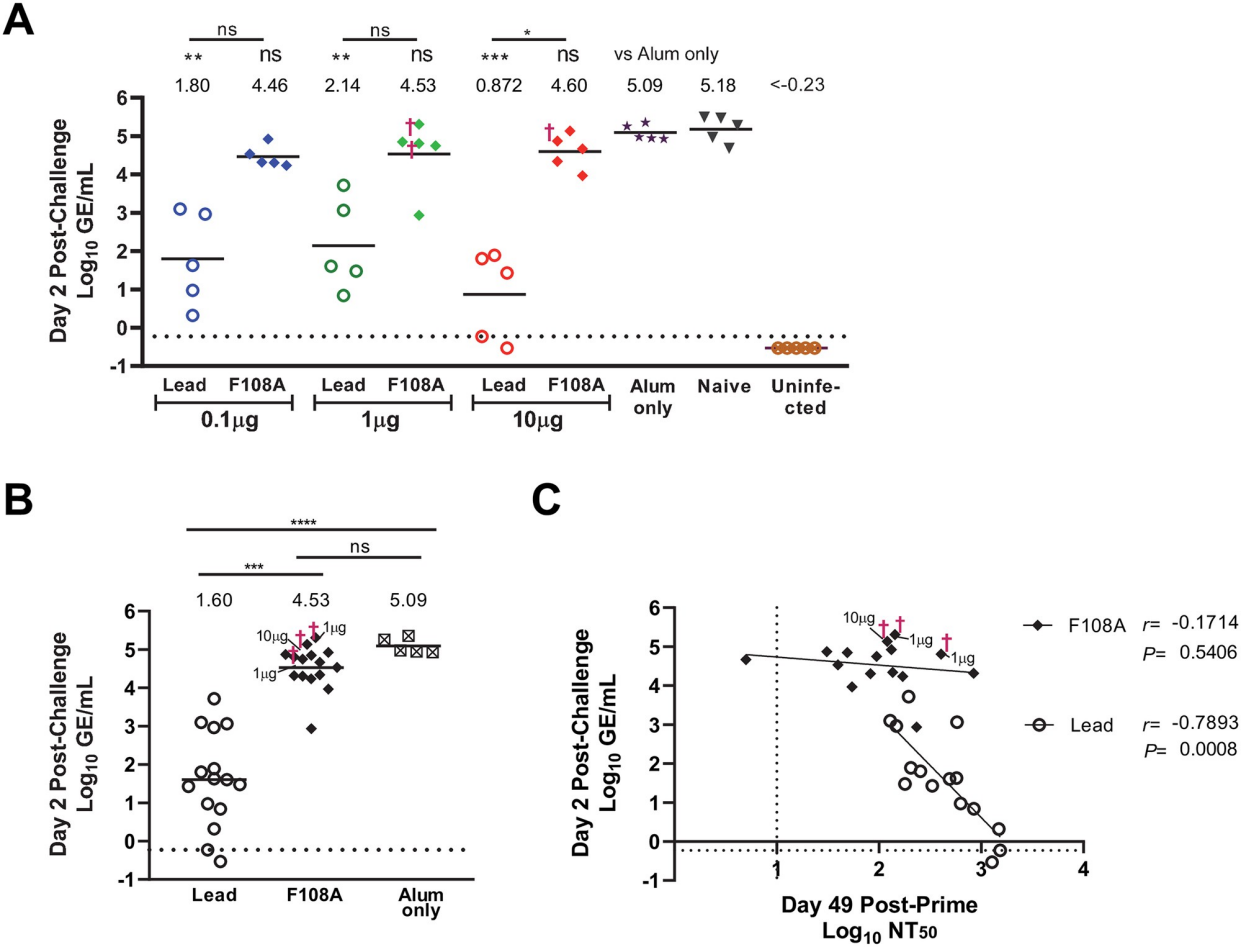
Immunization with lead, but not F108A VLP, significantly reduces RNAemia, which inversely correlates with nAb responses. Animals were challenged with 5,000 FFU of ZIKV strain (Nica 2–16) SC at Day 52 as depicted in Fig 3A. (A) Viral load in serum was measured on day 2 post-challenge by RT-qPCR. Each symbol represents the log_10_ GE/mL for an individual mouse, horizontal bars and values above each group show group means, significance values compared to the Alum-only group are just above group mean values, and the bars and significance levels on top were obtained by comparing lead and F108A groups. The dotted line is the limit of detection (LOD) of the assay set based on the lowest log GE/mL detected (0.59 GE/mL), and the symbol † denotes a mouse with a lethal infection. Measurements below the LOD were assigned a value of half the LOD. Statistical comparisons were performed using Kruskal-Wallis test followed by Dunn’s multiple comparisons tests (ns, not significant; *P<0.05; **P<0.01; ***P<0.001; ****P<0.0001). (B) Combined lead and combined F108A VLP groups were compared to the Alum-only group. Individual viral load values, assay LOD, and the symbol † designation are as in (A). (C) The relationships between NT_50_ and viral load (both log_10_ transformed) for the combined lead VLP groups and the combined F108A VLP groups were assessed by Spearman correlation analysis with resulting r and P values shown.

To measure protection against viremia after challenge, RT-qPCR was used to quantify ZIKV genomes in mouse sera on days 2 and 4 post-challenge (Figs 6A and S5, respectively). We found that on day 2 post-challenge, mice immunized with the lead VLP vaccine had statistically significant lower RNAemia (3- to 4-Log_10_ decreases) compared to the alum-only control mice, regardless of VLP dose (0.1μg, *P* = 0.0029; 1μg, *P* = 0.0046; and 10μg, *P* = 0.0005) (Fig 6A). In striking contrast, none of the F108A VLP groups showed significant differences in RNAemia levels compared to the Alum-only group (Fig 6A). RNAemia levels were lower in each of the lead VLP immunized dose groups relative to each of the F108A VLP groups immunized with the same VLP dose. The only statistically significant difference of RNAemia was between the 10μg dose group of lead and F108A (*P* = 0.036). By comparing the Alum-only group to the combined lead VLP and combined F108A groups we found that, overall, the F108A VLP groups had similar RNAemia levels compared to the Alum-only group (*P*>0.05), while the lead VLP groups had significant reductions in RNAemia compared to both the Alum-only group (*P*<0.0001) as well as the combined F108A groups (*P* = 0.0002) (Fig 6B). Together, these data show that protection against viremia was only observed in the lead VLP groups. Similar trends in RNAemia reduction were observed on day 4 post-challenge (S5 Fig). Only the 0.1 and 10μg dose groups showed significant reductions in RNAemia compared to the Alum-only group (*P* = 0.0135 and 0.0029, respectively), while the pairwise comparisons of lead and F108A groups at each dose were found to be significantly different only at 10μg (*P* = 0.0122).

For this study, we performed correlation analyses between pre-challenge NT_50_ titers and RNAemia levels in the combined lead VLP groups and combined F108A groups. The data in Fig 6C show a statistically significant inverse correlation between log_10_ GE/mL andlog_10_ NT_50_ in mice immunized with the lead VLP (*P* = 0.0008). However, the F108A VLP immunization was not significantly protective against RNAemia (Fig 6A and 6B), and there was no correlation between pre-challenge NT_50_ levels and day 2 RNAemia levels in the F108A VLP immunized mice (*P* = 0.54) (Fig 6C). Taken together, these challenge data showed that 1) only the lead VLP provided complete protection against ZIKV challenge and significant reductions in RNAemia compared to controls, 2) lead VLP provided significantly increased protection against weight loss compared to F108A groups, and 3) pre-challenge nAb levels in the lead VLP, but not F108A, groups showed a significant inverse correlation with viral RNA loads, suggesting a protective role for nAbs against the lead VLP.

### F108A VLP-induced immune sera enhance ZIKV infection *in vitro*

**Fig 7:**
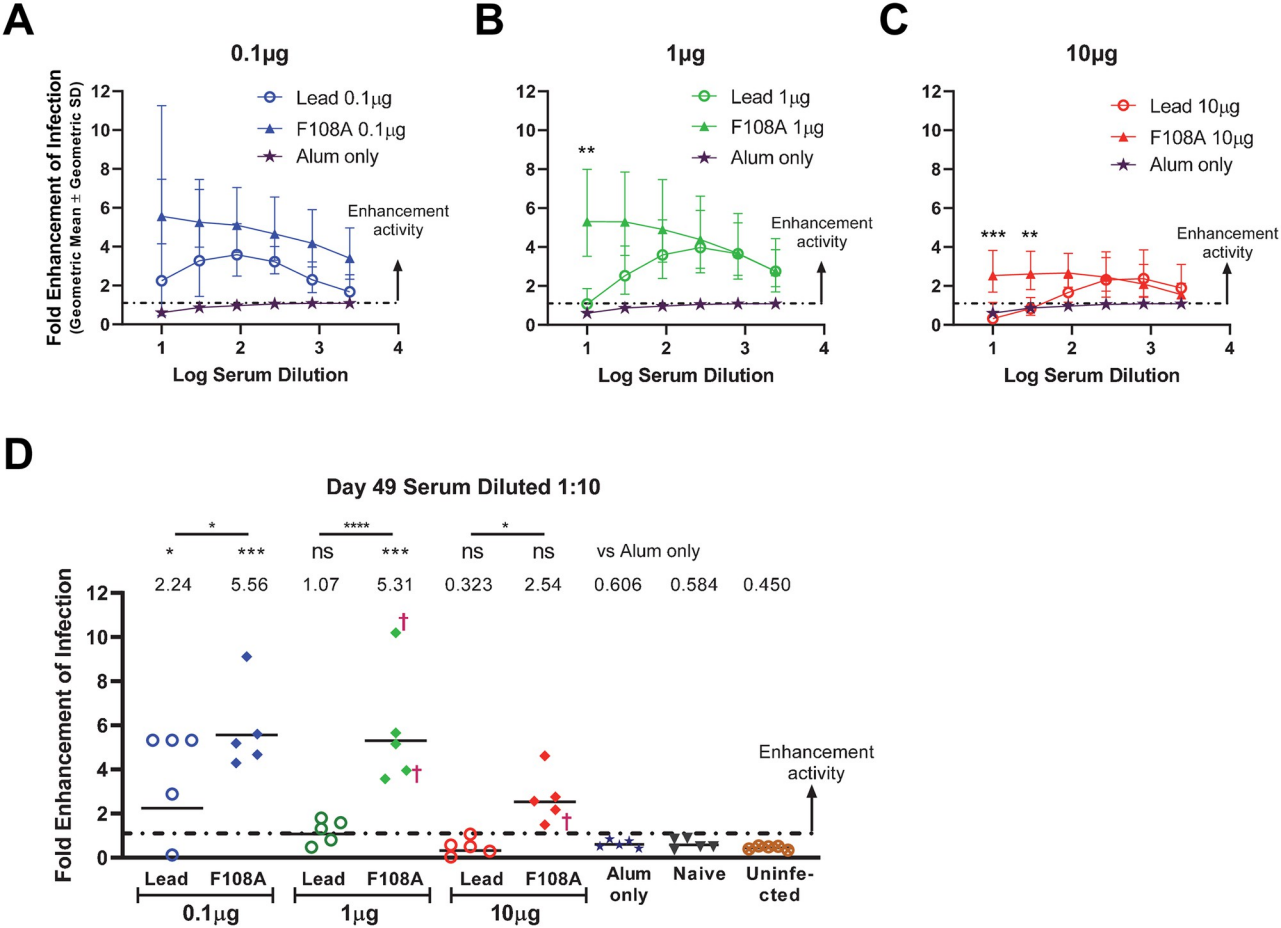
Ab-dependent Enhancement *in vitro* assay. (A-C) Fold enhancement of infection is represented for each serum dilution tested, ranging from 10-fold up to 2,430-fold. Fold-enhancement reflects the ratio of the percentage of cells infected with diluted serum-treated virus to medium only-treated virus. The calculated cutoff for ADE activity, shown by dot and dashed line, was calculated as the mean fold-enhancement values of the negative control groups (Alum-only, naïve, and uninfected) + 3 standard deviations (SD). Each symbol indicates the geometric mean of a dose group (5 mice), and error bars represent geometric SD. The three dose groups, 0.1μg (A), 1μg (B) and 10μg (C), were separated into different graphs in which lead and F108A VLP-immunized mice are compared. The Alum-only group is shown in each panel for comparative purposes. Statistical comparisons of enhancement data were performed at each dose using one-way ANOVA test followed by pairwise Sidak’s multiple comparisons between lead and F0108A groups at each serum dilution (*P<0.05; **P<0.01; ***P<0.001; ****P<0.0001; ns, not significant). (D) Fold-enhancement of infection of cells infected with the 10-fold serum dilution. Each symbol represents the geometric mean of two independent measurements for an individual animal. Horizontal bars represent the geometric mean titer of each group. The symbol † denotes a mouse that succumbed after challenge. Statistical comparisons were performed using ANOVA test followed by Dunnett’s multiple comparisons tests with significance levels denoted as in (A-C) above. All VLP groups were compared to the Alum-only group. Additional pairwise comparisons were performed between titers of lead and F108A groups at each dose with significance level for each comparison shown above each bar.

An *in vitro* assay was performed to assess ADE of ZIKV infection using sera from lead or F108A VLP immunized mice. Serial dilutions of post-boost (Day 49) serum samples were incubated with ZIKV Nica 2–16 and used to infect FcγR expressing K562-r cells. Infected cells were quantified using a flow cytometry-based assay and the fold-enhancement of infection was calculated as the percentage of cells infected with a serum dilution/virus mixture relative to cells infected with medium alone/virus mixture. A threshold for positive ADE activity was also set as the mean fold-enhancement of infection value of mice from negative control groups (Alum-only, Naïve and Uninfected) plus 3 SD. We found that sera from mice immunized with 0.1μg VLP had geometric mean values above the enhancement threshold and that, at each dilution tested, the values for F108A VLP were not significantly different from those of the lead VLP group (Fig 7A). In contrast, a significant increase in ADE activity was observed for F108A VLP compared to lead VLP-specific immune sera for the 1μg dose group at 1:10 serum dilution (*P* = 0.0014) (Fig 7B) and for the 10μg dose group at 1:10 and 1:30 serum dilutions (*P* = 0.0066 and *P* = 0.0335, respectively) (Fig 7C). We also observed that when the immune sera from the lead VLP-immunized mice were serially diluted in the assay, fold enhancement values increased progressively and then decreased at the highest dilutions forming a bell-shaped curve (Fig 7A–7C).

Since a 1:10 serum dilution is closest to the physiological concentrations (i.e., undiluted), this dilution was used in the assay to show differences between individual serum samples (Fig 7D). All 15 of the mice immunized with F108A VLP showed values above the calculated threshold for ADE activity. A dose-dependent effect of ZIKV-specific ADE activity was observed for mice immunized with the lead VLP. Four out of 5 mice showed ADE activity in the 0.1μg dose group, 3 out of 5 in the 1μg dose group and none in the 10μg dose group. Pairwise comparisons were made between each VLP group and the Alum-only group as well as between lead VLP and F108A VLP groups at each VLP dose. ADE was significantly increased, as compared to sera from the Alum-only group, when using sera from mice immunized with 0.1μg of lead VLP (*P* = 0.020) as well as with sera from immunizations with 0.1μg and 1μg of F108A VLP (*P* = 0.0001 and *P* = 0.0002, respectively). Most strikingly, the serum ADE values for F108A VLP groups were significantly higher than their respective lead VLP groups at each dose level (*P* = 0.0348, *P*<0.0001, and *P* = 0.0348 for the 0.1μg, 1μg, and 10μg dose group, respectively), indicating that the F108A VLP induces higher levels of infection-enhancing Abs than the lead VLP candidate. Together, these results provide *in vitro* evidence that the F108A VLP, in addition to eliciting lower nAb levels than the lead VLP, induced significantly higher levels of ZIKV infection enhancing Abs that further increased with decreasing VLP dose.

## Discussion

We report herein a ZIKV VLP vaccine that induced robust protective nAb responses in mice. Protection was based on reduction of viral genome copies, protection against weight loss, and survival. We consider this robust protection. We note that while the observed 3- to 4-Log_10_ reductions in mean genome copy numbers were significant, we did not specifically address levels of infectious virus and whether low RNA copy numbers indicated the presence of infectious virus. Previous mouse and NHP studies also supported development of this VLP vaccine candidate [61, 62]. In our NHP study, we addressed viremia using our lead VLP vaccine followed by a ZIKV challenge [62]. Using the less sensitive plaque assay, relative to RT-qPCR, as a readout, we detected no viremia following viral challenge. Whether our vaccine is capable of achieving sterilizing immunity requires further investigation. However, it should be noted that attaining sterilizing immunity following vaccination is an important goal but difficult to realize. As witnessed with the recent Moderna and Pfizer vaccines for COVID-19 disease, success was due to prevention of severe disease and death while breakthrough infections were evident [76–78].

It is generally accepted that highly efficacious and protective immune responses rely, in part, on CD4+ T cells that produce cytokines that activate and maintain CD8+ T cells that clear virus-infected cells as well as B cells that produce Abs that can prevent receptor attachment and cell entry. Although not the focus of this study, our ZIKV VLP vaccine likely induces sufficient CD4+ T cell responses specific for the ZIKV prM and E structural proteins, as helper T cell responses were presumably required for induction of the ZIKV-specific nAb responses that we observed following VLP immunization. Although recombinant protein vaccines and/or VLP-based vaccines may induce some CD8+ T cell responses, they would likely not be the optimal vaccine platform for this goal [79]. Generally, live-attenuated vaccines, viral vectors, and nucleic acid platforms would be anticipated to induce robust CD8+ T cells. CD8+ T cells are thought to play a protective role in ZIKV infection based on studies in animal models, but there is less available data in humans [80]. Grifoni and colleagues elucidated in-depth characterization of human CD8+ T cells responding to ZIKV infection that are characterized by a polyfunctional IFNγ signature with upregulation of TNFα and related activation markers [81]. Thus, a live attenuated vaccine approach for ZIKV vaccine development to induce both humoral and cellular immune responses, such as in the case of the licensed yellow fever (YF) vaccine [82–84], would seem a valid approach, although there would be significant safety concerns.

Studies have shown that one dose of YF vaccine may provide at least 35 years of immunity with detectable Abs up to 40 years and a YF-specific memory T cell pool for at least 18 years post-vaccination [85]. In the case of the licensed recombinant live attenuated virus vaccine, Dengvaxia, which utilizes the YF nonstructural protein backbone and DENV-specific prM and E structural proteins, children receiving the vaccine and who had never experienced prior DENV infection were more susceptible to hospitalization and severe disease when exposed to a subsequent DENV infection [50–58]. It is suggested that an ideal DENV vaccine that induces long-lasting homotypic nAb responses to all four serotypes in all age groups and elicits CD8+ T cell responses to intracellular (nonstructural) antigens of DENV to increase efficacy of the vaccine would be advantageous [86]. However, in the case of ZIKV vaccine development, a live-attenuated vaccine would be difficult to develop due to potential harm to the developing fetus. We have focused our ZIKV vaccine development on a virus-like-particle approach to induce protective nAb. This approach of inducing protective nAbs is, in part, supported by the efficacy of inactivated flavivirus vaccines such as tick-borne encephalitis (TBE) and Japanese encephalitis virus (JEV), which were licensed based on protective Abs as the primary immune correlate of protection. The YF 17D vaccine developed in the 1930s by Max Theiler and colleagues is one of the oldest live-attenuated vaccines, and the level of nAbs required for protection is a Log_10_ neutralizing index of 0.7, or PRNT_50_ value of 1:20 to 1:40 [82, 83, 87, 88]. Regarding the inactivated vaccine for JEV, IXIARO, a PRNT_50_ titer of at least 10 has been shown to correlate with protection against development of JE disease in humans [89, 90]. The TBE vaccine Ticovac was recently approved (August 2021 FDA; February 2022 ACIP) for use in US travelers but has been available in Europe for over 20 years. An early study indicated a level of 125 ELISA units is sufficient for protection against TBE disease in mice [91], while a more recent CDC presentation [92] specified that a nAb titer ≥10 was used in vaccine studies to indicate protection.

In summary, our VLP vaccine approach induced robust nAb responses, protected mice against high viral loads, weight loss, and death; thus, these data support further ZIKV VLP vaccine development. It is however, noted that development of an ‘ideal’ vaccine using a different vaccine platform and/or additional viral antigens with the goal of inducing highly protective humoral and cellular responses is desirable but outside the scope of these studies.

Analysis of mAbs derived from ZIKV-infected donors revealed that the anti-viral humoral response is directed primarily to the E protein [14, 93–96]. To this end, the vaccine development strategy described herein is based on VLPs displaying to the immune system E protein comparable to virus encountered during endemic or pandemic spread. VLPs are an ideal platform for generation of ZIKV vaccines as they can be produced from prM/M and E proteins that undergo pH-induced rearrangements and conformational changes similar to the wild-type virus [97]. In previous studies, we evaluated the lead ZIKV VLP candidate where we observed yields of column purified VLP in the range of 1-2mg/L following *in vitro* transfection of prM/M and E DNA in HEK293T cells [61, 62]. In order to potentially increase VLP yields, we adopted a strategy from Urakami and colleagues, who reported that mutating the E protein at position F108A in the FL of DENV-1 increased yields 16-fold when producing DENV-1 VLP in mammalian cells [64]. Although increasing VLP production yield was desirable, especially for downstream scalability, it was important that any modifications did not come at the expense of VLP immunogenicity and efficacy. Initial production runs of the F108A ZIKV VLP indicated that the yield was approximately only 2-fold higher than the lead candidate. Evaluation of both VLP particles by SDS-PAGE and Western blot revealed that the F108A particle lacked detectable prM processing by furin relative to the lead candidate. However, transmission electron micrographs indicated that particles were comparable in size and appearance, at least at the resolution achieved. BLI analysis using a panel of characterized ZIKV binding mAbs demonstrated that Ab binding to F108A VLP, relative to lead, was mostly reduced in mAbs that bind quaternary epitopes on EDII (mAbs ZIKV-117 and LM-081) and on EDIII outside of the lateral ridge (LM-081), while mAbs that bind to the lateral ridge of EDIII (ZIKV-116 and ZV-67) were only slightly reduced.

Although binding Ab levels induced by the F108A VLP were similar to lead VLP, neutralization activity was reduced. Following ZIKV challenge, the F108A VLP-immunized groups that exhibited a trend in weight loss had a significantly higher RNAemia compared to the lead VLP-vaccinated groups, and 3 of 15 mice succumbed to infection. Interestingly, nAb levels elicited by F108A VLP immunizations did not correlate with RNAemia in serum, in contrast to the lead VLP-induced nAbs, which were inversely correlated. Finally, an evaluation of *in vitro* enhancement of ZIKV infection indicated that F108A immune sera had a higher propensity to enhance ZIKV infection, especially at the intermediate 1μg and high 10μg doses.

A single amino acid residue mutation in E prevented prM from being efficiently cleaved on ZIKV VLPs. This modification appears to have prevented this vaccine candidate from inducing a protective response in mice against ZIKV challenge. Improving our understanding of how the structure of the VLP’s surface contributes to the induction of a protective immune responses should aid future flavivirus vaccine antigen designs [98]. Based on these immunogenicity and efficacy data, the lead vaccine candidate was shown to be more suitable for vaccine development versus the F108A VLP candidate. The threshold of prM cleavage that is adequate to produce a safe and protective ZIKV vaccine remains unknown, and we believe that there is an unmet need to set this criterion. Importantly, a previous study showed that significantly reducing the amount of unprocessed prM on the surface of an immature DENV serotype-2 VLP vaccine candidate conferred higher protection to all four DENV serotypes compared to its immature VLP counterpart [99]. Measures of vaccine efficacy in flavivirus challenge models are dependent on the levels of prM processing of both the immunogen and the challenge virus. The levels of prM processing and maturity of the challenge virus used in the mouse efficacy experiments and ZIKV used in the ADE assay are unknown but would be important to address in future studies to further examine the role of particle maturity. We note that for the studies described herein, virus passage numbers were kept to a minimum since serial *in vitro* passage of DENV-1 was shown to decrease levels of virion maturation [100]. Partially mature reporter-Luc virus used in the present nAb assays and mature reporter-Luc virus produced in the presence of furin were previously shown to be equally neutralized by well-characterized nAbs [74].

Interestingly, the apparent difference in maturation states between the lead and F108A ZIKV VLPs does not seem to correlate with the proportions of particle sizes. Particle diameters ranging from approximately 30nm to 50nm were observed in both lead and F108A purified VLP preparations. The 50nm particle size is comparable to the T = 3 symmetry, usually found for ZIKV *in vivo*, whereas the 30nm particles may correspond to T = 1 symmetry [101]. Particle size heterogeneity has been studied previously for flavivirus VLPs, but conclusions are mixed regarding a possible link to particle maturity and immunogenicity. While Ferlenghi and colleagues generated an immunogenic TBEV VLP preparation with 90% mature small particles (31 nm) [102], Ohtaki and colleagues observed that their larger particles (40-50nm) were more immunogenic and mature than the smaller particles (20-30nm) for WNV VLPs [103]. Shen and colleagues propose that the structure of their smaller DENV VLP particles with T = 1 symmetry might be advantageous for inter-dimeric epitope accessibility on the E protein [99]. Moreover, the absence of capsid in the VLP constructs may explain the observed particle size heterogeneity more so than particle maturity. Tan and colleagues’ work on ZIKV structure supports the notion that capsid plays an important part in the virus assembly process and sustains its structure [104].

As mentioned above, it has been extensively documented that secondary infection with DENV from a heterologous serotype increases the risk for severe DENV disease. One of the root causes of this effect has been linked to the presence of non-neutralizing or poorly neutralizing Abs in affected individuals. As generation of cross-reactive Abs between ZIKV and DENV1-4 following infection with either virus is highly prevalent due to the similarity of these viruses’ surface epitopes [105], checking for signs of exacerbated disease outcome following secondary infection with either virus would appear to be of utmost importance. Most recently, evidence has been put forward as to the implication of preexisting ZIKV Abs increasing cases of symptomatic and severe DENV-2 infection in humans [35]. The reverse, DENV antibodies enhancing ZIKV infection, has been shown as well *in vitro* [106]. Less is known about the risk of enhanced ZIKV infection following ZIKV-priming. However, when studying the protective potential of purified polyclonal ZIKV IgG, Pinto and colleagues concluded that careful calculation of dosage is needed upon immunization as there exists a “window of caution” when the neutralizing ability of the administered antibodies is dampened by enhancement of viral infection. Although immunization with our lead ZIKV VLP-induced Abs with high neutralization capacity and absence of ADE was observed at low serum dilutions, we cannot rule out the possibility that individuals may eventually over time develop Abs within the said “window of caution” due to waning immunity. Thus, safety and efficacy are key criteria in the development of a flavivirus vaccine and generation of non-neutralizing Abs or Abs at sub-neutralizing concentrations should be prevented, particularly when Abs to conserved epitopes can cross-react with other flaviviruses and cause ADE [107]. Indeed, Abs directed to the highly conserved FL peptide or prM do not efficiently neutralize infection but may render non-infectious immature virions highly infectious and thus be causative agents of ADE [37, 108]. Here we studied ADE *in vitro* using K562 FcγR-expressing cells. K562 cells have been used in the past as a tool to evaluate ZIKV ADE *in vitro* [106, 109, 110], as well as ADE in other flavivirus infections [64]. Monocyte-derived macrophages also may be used as reporter cells for ADE, however, Pinto and colleagues have shown that similar ZIKV ADE levels were obtained when using either cell type over a similar range of antibody concentrations [109]. The same group also demonstrated that low concentrations of purified ZIKV IgG drove enhanced ZIKV *in vivo* similar to the effect seen *in vitro* on K562 cells and monocyte-derived macrophages.

We observed that Abs induced by the F108A VLP induced a lower percentage of binding antibodies that were neutralizing (Fig 4C and 4D) and that enhanced ADE (Fig 7) compared to the lead candidate. It is possible that the poor maturity of this VLP led to antibodies that promoted ADE. A similar finding was described for a ZIKV DNA vaccine in which the parent construct was further modified to increase the yield of *in vitro* secreted virus-like subparticles (SVPs) [24]. The higher-yield construct was found to be less mature and less protective in NHPs than the parent. Further characterization of nAb responses suggested that protection was associated with better neutralization against mature forms of the virion rather than total nAb levels. Consistent with these findings, we observed that the F108A ZIKV immature particle-induced immune sera also had reduced neutralizing ability compared to the lead ZIKV VLP candidate, which consisted of relatively more mature particles. Several groups developing flavivirus vaccines have observed that more mature particles resulting from increased prM processing improve nAb responses and/or protect better than the less mature counterparts [99, 103, 111, 112]. To circumvent the issue posed by incomplete prM processing, investigators have gone to the extent of developing vaccines directed only against the DENV envelope [113–115]. However, a study by Rodenhuis-Zybert in which human DENV convalescent sera was analyzed suggests that the titer of prM IgG may not be a good predictor of disease severity [116]. We acknowledge that although our studies indicate that the F108A vaccine candidate had a higher capacity to induce *in vitro* enhancement, this observation can’t be used as a predictor of *in vivo* enhancement in vaccinated individuals exposed to ZIKV infection. Importantly, our *in vitro* observation of enhancement together with decreased immunogenicity and efficacy is sufficient to halt vaccine development of the F108A ZIKV vaccine candidate.

In summary, the strategy of introducing a mutation in the FL (F108A) to increase VLP yields following prM/M and E DNA transfection in HEK293T cells was minimally successful. Only an approximate 2-fold increase in purified VLP production was realized. Additionally, the F108A particle also induced a significantly less efficacious immune response, a detriment to vaccine development. This loss in efficacy is likely due in large part to generating a relatively less mature particle, which in turn induced lower nAb levels and higher levels of Abs that promoted virus infectivity. In fact, there was no correlation between nAbs induced by the F108A particle and level of RNAemia. These studies support the importance of a critical evaluation of particle and virus maturity in flavivirus vaccine development.

## Supporting information

S1 Dataset Complete dataset for all plots and statistical analyses. Excel.xlsx file with a tab named for each figure panel (e.g., Fig 2C) that contains the corresponding dataset for that panel.
(XLSX)

S1 Fig mAb binding to F108A and lead VLP measured by Biolayer Interferometry. Symbols represent each measurement, connecting lines show the 4PL regression curve (for ZV-48 F108A, there were only 3 detectable points so no regression could be performed), dotted lines indicate 95% CI bounds for the lead regression, and R squared values are shown for each lead regression.
(TIF)

S2 Fig Coomassie and anti-ZIKV prM Western blot analysis of the VLP used to immunize AG129 mice. Purified F108A VLP fractions (A) and lead VLP fractions (B) collected from a chromatography system were run on an SDS-PAGE gel and stained by Coomassie to identify the VLP proteins and control for purity. The VLP containing fractions bracketed above were then pooled to prepare the F108A ZIKV VLP and lead VLP material. (C) Pooled material was run on an SDS-PAGE gel, and maturity of particles was tested by staining with an anti-ZIKV prM Ab (Genetex, cat#gtx133305).
(TIF)

S3 Fig ZIKV serum neutralizing antibody titers after priming immunization with lead or F108A VLP (Day 21 of study). Serum samples obtained prior to boosting were analyzed by luciferase-expressing ZIKV reporter particle to determine each 50% neutralization titer (NT_50_). Symbols represent the mean from two assay replicate values for each serum, bars represent GMT, and the dotted line represents the assay LOD. Titers below LOD were assigned a value one-half the LOD (5) for graphing and statistical analysis. Statistical comparisons were using Kruskal-Wallis test followed by Dunn’s multiple comparisons tests between 1) the Alum-only group vs. each VLP group and between 2) the lead and F108A VLP groups at each dose level (9 comparisons total). Significance levels are shown by asterisks as follows, *P<0.05, **P<0.01, ***P<0.001, ****P<0.0001, and ns (not significant).
(TIF)

S4 Fig Weight change comparisons following ZIKV challenge of immunized mouse groups and an unchallenged (Uninfected) group vs. the Alum-only control group. Statistical significances of percentage weight changes between groups on each day post-challenge were obtained by one-way ANOVA followed by Dunnett’s multiple comparisons tests vs. the Alum-only group.
(TIF)

S5 Fig Viral load in serum was measured on day 4 post-challenge by RT-qPCR. Each symbol represents the log_10_ GE/mL for an individual mouse, horizontal bars and values above each group show group means, significance values vs. the Alum-only group are just above group mean values, and the bars and significance levels at the top are from comparing lead and F108A groups. The dotted line is the limit of detection (LOD) of the assay set based on the lowest log GE/mL detected (0.59 GE/mL). Measurements below the LOD were assigned a value of half the LOD. Statistical comparisons were performed using Kruskal-Wallis test followed by Dunn’s multiple comparisons tests (ns, not significant; *P<0.05; **P<0.01; ***P<0.001; ****P<0.0001). All VLP groups were compared to the Alum-only group, and the lead and F108A groups were compared at each dose level.
(TIF)
