# Supplementary figures and images for: Zika virus-like particle vaccine fusion loop mutation increases production yield but fails to protect AG129 mice against Zika virus challenge
Source: PLoS Negl Trop Dis. 2022 Jul 6;16(7):e0010588. doi: 10.1371/journal.pntd.0010588 (PMC9292115; doi:10.1371/journal.pntd.0010588)

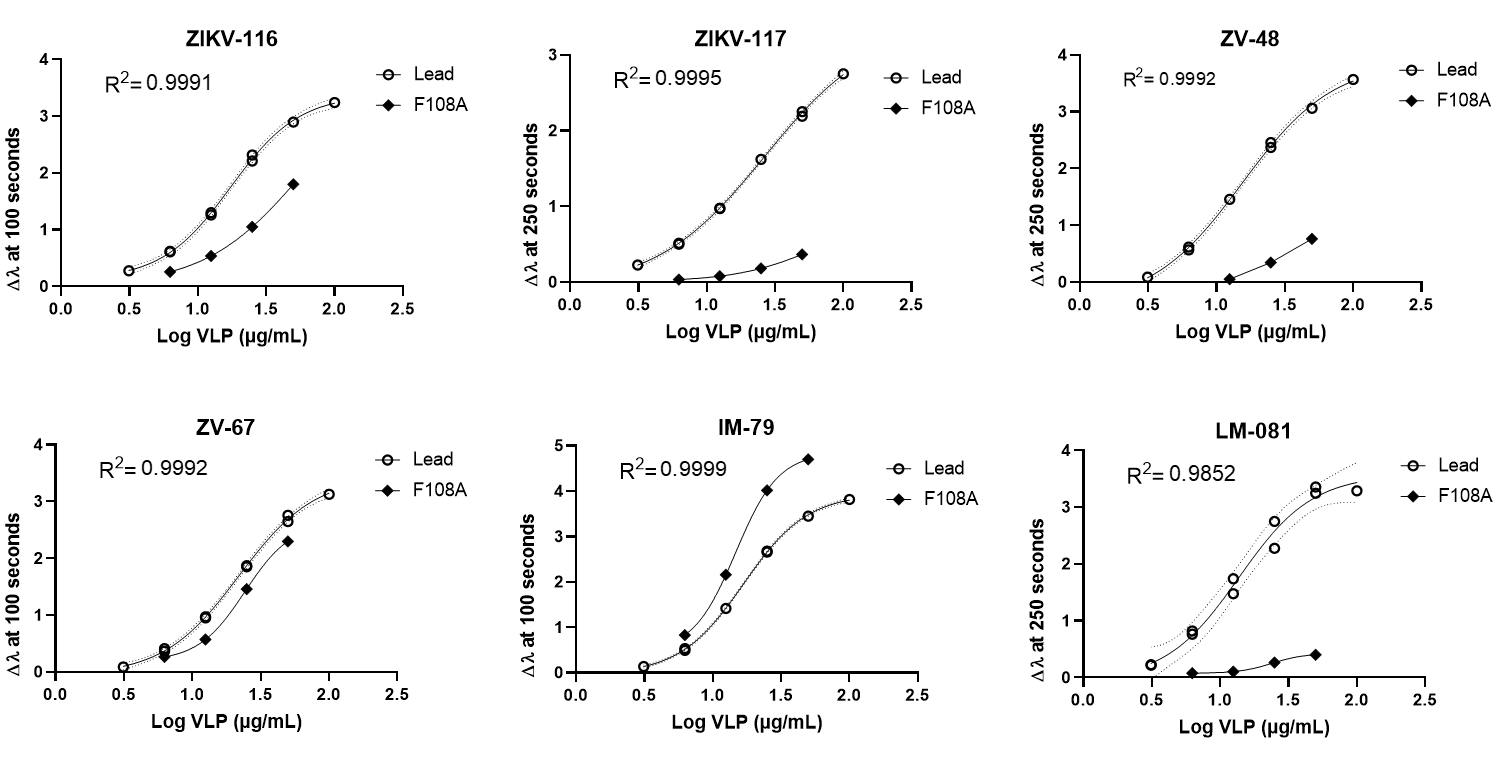

Supplement: S1 Fig — Symbols represent each measurement, connecting lines show the 4PL regression curve (for ZV-48 F108A, there were only 3 detectable points so no regression could be performed), dotted lines indicate 95% CI bounds for the lead regression, and R squared values are shown for each lead regression. (TIF) [file pntd.0010588.s002.tif]

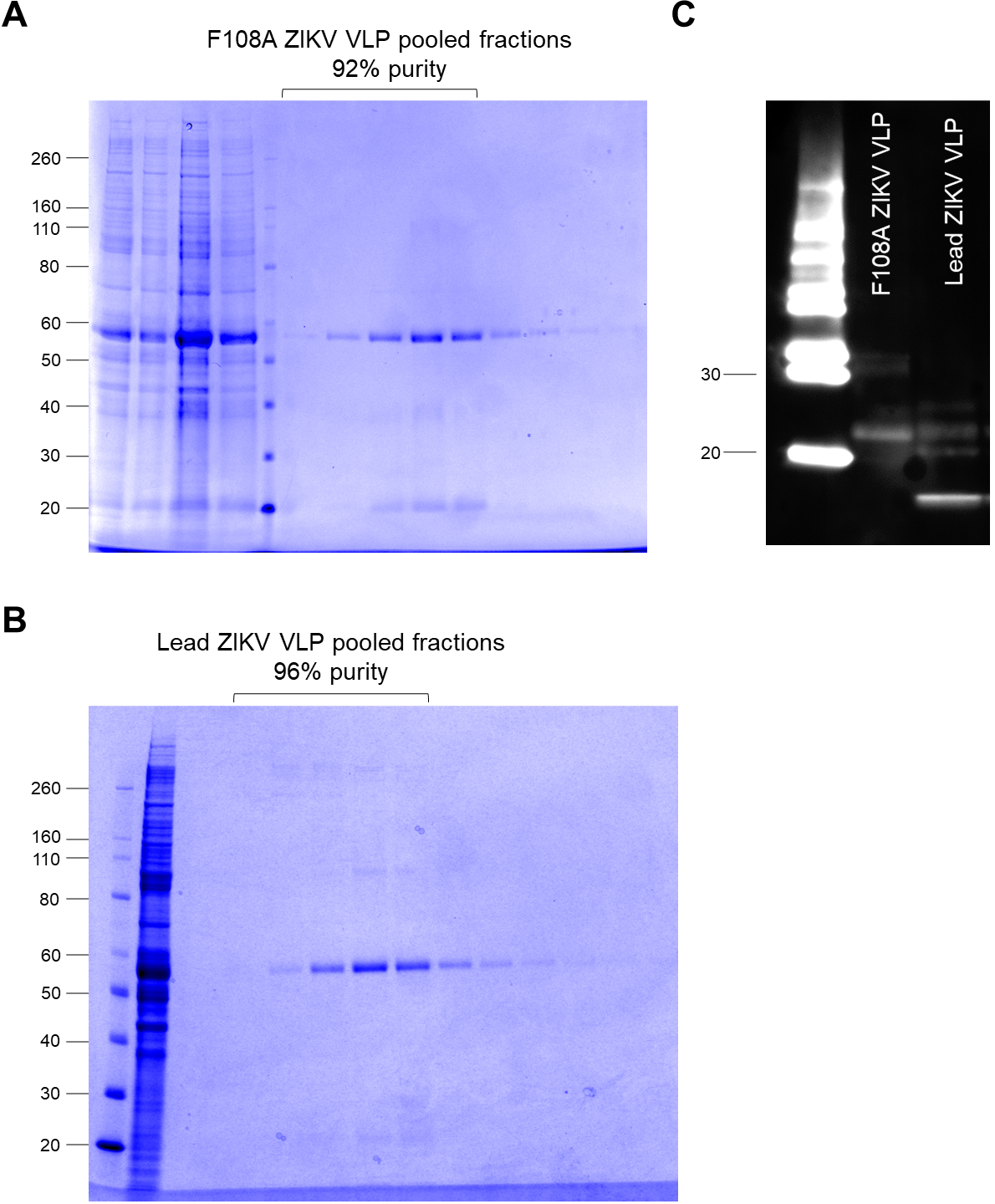

Supplement: S2 Fig — Purified F108A VLP fractions (A) and lead VLP fractions (B) collected from a chromatography system were run on an SDS-PAGE gel and stained by Coomassie to identify the VLP proteins and control for purity. The VLP containing fractions bracketed above were then pooled to prepare the F108A ZIKV VLP and lead VLP material. (C) Pooled material was run on an SDS-PAGE gel, and maturity of particles was tested by staining with an anti-ZIKV prM Ab (Genetex, cat#gtx133305). (TIF) [file pntd.0010588.s003.tif]

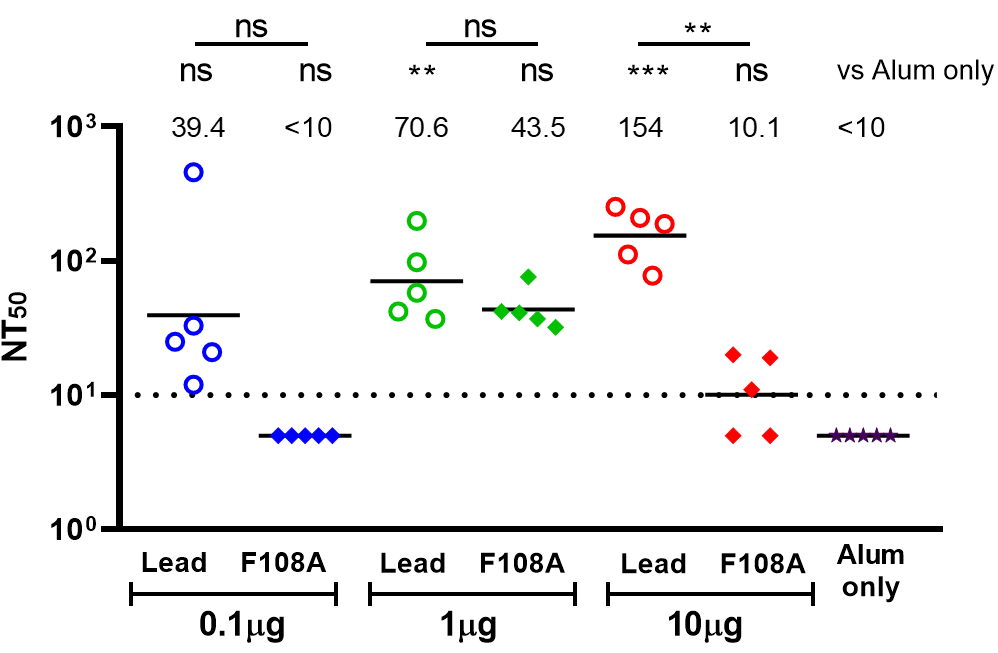

Supplement: S3 Fig — Serum samples obtained prior to boosting were analyzed by luciferase-expressing ZIKV reporter particle to determine each 50% neutralization titer (NT50). Symbols represent the mean from two assay replicate values for each serum, bars represent GMT, and the dotted line represents the assay LOD. Titers below LOD were assigned a value one-half the LOD (5) for graphing and statistical analysis. Statistical comparisons were using Kruskal-Wallis test followed by Dunn’s multiple comparisons tests between 1) the Alum-only group vs. each VLP group and between 2) the lead and F108A VLP groups at each dose level (9 comparisons total). Significance levels are shown by asterisks as follows, *P<0.05, **P<0.01, ***P<0.001, ****P<0.0001, and ns (not significant). (TIF) [file pntd.0010588.s004.tif]

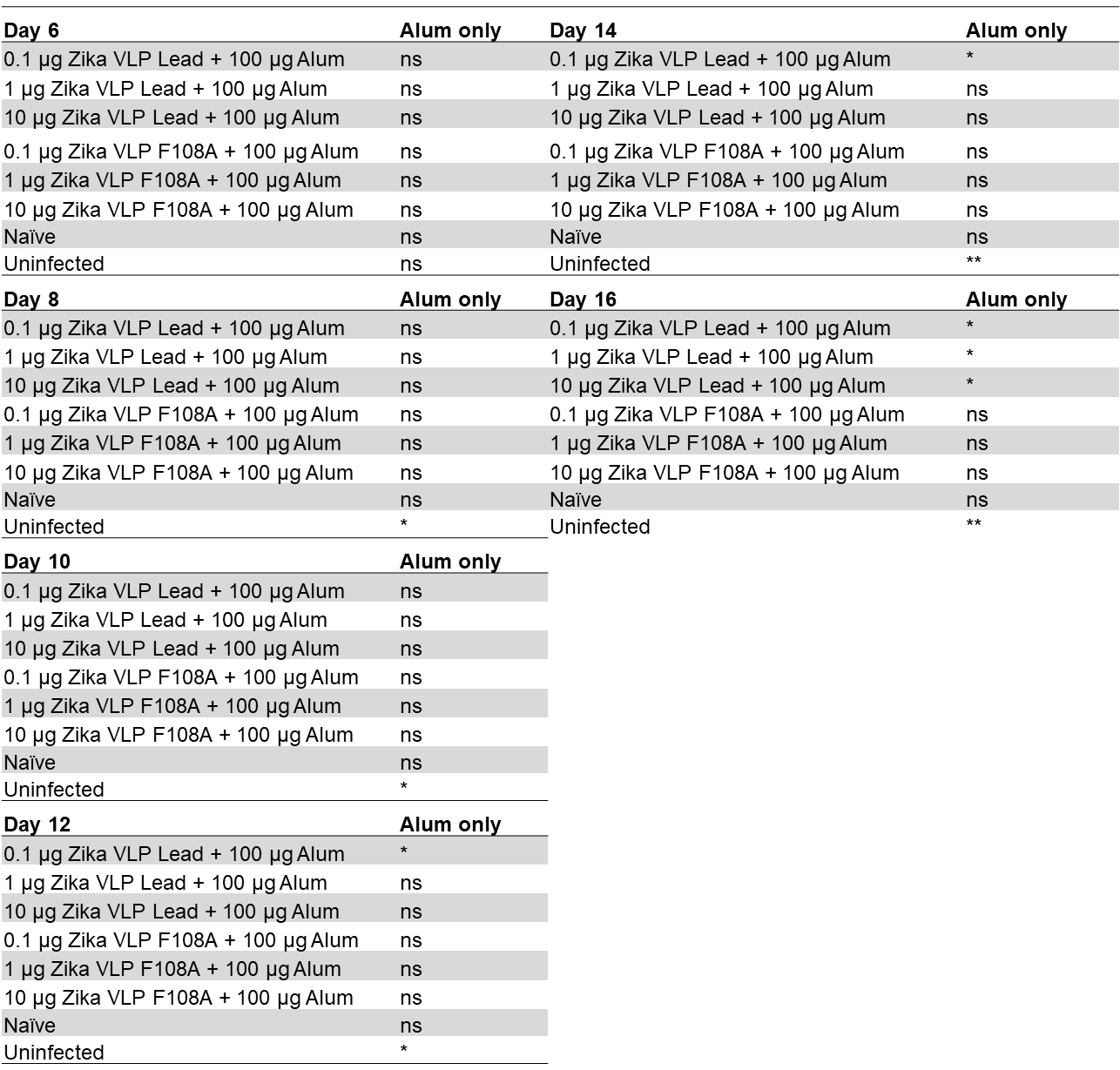

Supplement: S4 Fig — Statistical significances of percentage weight changes between groups on each day post-challenge were obtained by one-way ANOVA followed by Dunnett’s multiple comparisons tests vs. the Alum-only group. (TIF) [file pntd.0010588.s005.tif]

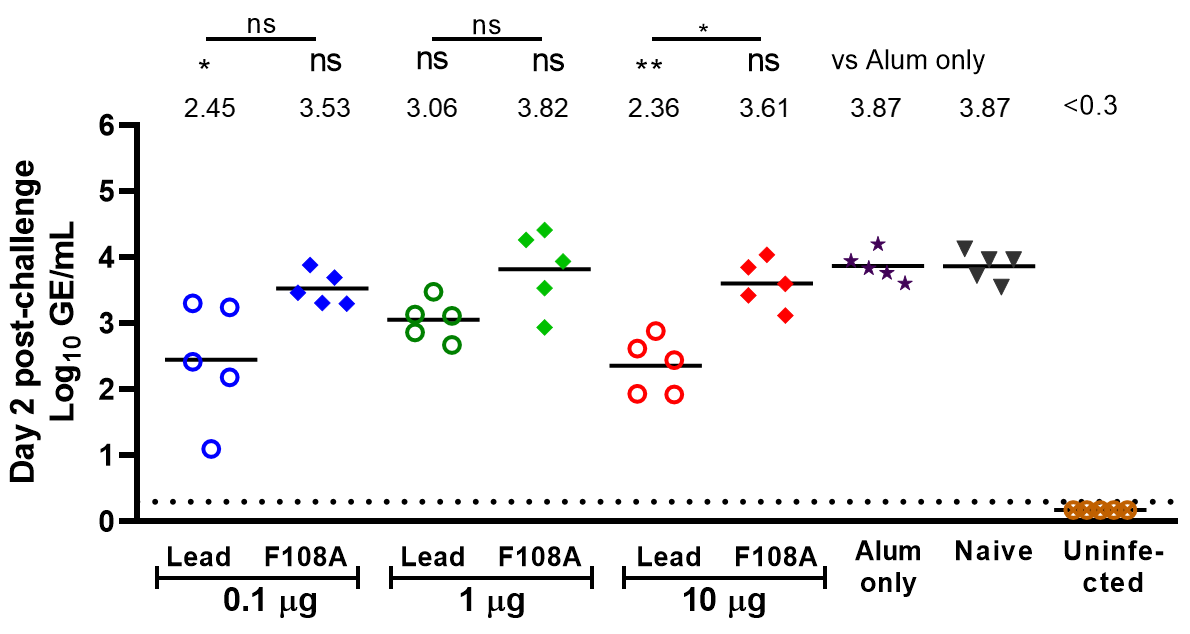

Supplement: S5 Fig — Each symbol represents the log10 GE/mL for an individual mouse, horizontal bars and values above each group show group means, significance values vs. the Alum-only group are just above group mean values, and the bars and significance levels at the top are from comparing lead and F108A groups. The dotted line is the limit of detection (LOD) of the assay set based on the lowest log GE/mL detected (0.59 GE/mL). Measurements below the LOD were assigned a value of half the LOD. Statistical comparisons were performed using Kruskal-Wallis test followed by Dunn’s multiple comparisons tests (ns, not significant; *P<0.05; **P<0.01; ***P<0.001; ****P<0.0001). All VLP groups were compared to the Alum-only group, and the lead and F108A groups were compared at each dose level. (TIF) [file pntd.0010588.s006.tif]
